# Psychiatric Morbidity Following Intestinal Infectious Diseases: A Nationwide Cohort Study in South Korea

**DOI:** 10.1002/smi.70103

**Published:** 2025-08-29

**Authors:** Chaeyoon Kang, Seung Won Lee, Hohyun Jung, Youngoh Bae

**Affiliations:** ^1^ Sungkyunkwan University School of Medicine Seoul Republic of Korea; ^2^ Department of Precision Medicine Sungkyunkwan University School of Medicine Suwon Republic of Korea; ^3^ Personalized Cancer Immunotherapy Research Center Sungkyunkwan University School of Medicine Suwon Republic of Korea; ^4^ Department of Artificial Intelligence Sungkyunkwan University Suwon Republic of Korea; ^5^ Department of Family Medicine Kangbuk Samsung Hospital Sungkyunkwan University School of Medicine Seoul Republic of Korea; ^6^ Department of Statistics Sungshin Women's University Seoul Republic of Korea; ^7^ Data Science Center Sungshin Women's University Seoul Republic of Korea; ^8^ Department of Neurosurgery Korean Armed Forces Capital Hospital Seongnam Republic of Korea

**Keywords:** cohort studies, gastrointestinal diseases, mental disorders, risk factors, South Korea

## Abstract

Intestinal infectious diseases (IIDs), typically considered self‐limiting, may exert lasting effects on mental health. This nationwide retrospective cohort study investigated the association between recurrent IID and the subsequent development of psychiatric disorders in South Korea. Using data from the National Health Insurance Service‐National Sample Cohort (2002–2013), adults with three or more IID diagnoses were matched to controls without IID by age, sex, and health screening year. Eight psychiatric outcomes were examined: depressive disorder, bipolar disorder, anxiety disorder, obsessive‐compulsive disorder (OCD), adjustment disorder, organic mental disorders, schizophrenia, and alcohol use disorder. Patients with recurrent IID showed significantly increased risks for depressive disorder (adjusted hazard ratio [aHR], 2.14), bipolar disorder (aHR, 1.80), anxiety disorder (aHR, 2.20), OCD (aHR, 3.84), adjustment disorder (aHR, 2.33), and organic mental disorders (aHR, 1.67). Psychiatric risks were disproportionately higher among younger individuals and male patients. A dose‐dependent increase in psychiatric risk was observed with higher IID frequency. No significant associations were found for schizophrenia or alcohol use disorder in the overall analysis, although subgroup analyses revealed elevated risks with higher IID exposure. These findings suggest that recurrent IID may contribute to psychiatric morbidity via gut‐brain axis disruption and systemic inflammation. Clinical attention to mental health following IID episodes, particularly in vulnerable populations, may be warranted.

## 1 Introduction

Intestinal infectious diseases (IIDs) are characterised by inflammation of the gastrointestinal tract caused by pathogens such as viruses, bacteria, or parasites, and are commonly associated with symptoms including diarrhoea, abdominal pain, vomiting, and fever (Graves 2013). Although the global incidence of IIDs has shown a gradual decline, they remain highly prevalent, with an estimated 4.67 billion cases reported annually worldwide (Ferrari et al. 2024). IIDs can negatively affect daily functioning and quality of life and continue to impose a considerable burden on both communities and healthcare systems, not only in developing countries but also in high‐income settings (Tam et al. 2011; Choe et al. 2018). Furthermore, accumulating evidence suggests that the impact of IIDs may extend beyond transient gastrointestinal symptoms, contributing to the development of psychiatric disorders (Yu et al. 2022).

In recent years, growing attention has been directed towards the gut‐brain axis, a bidirectional communication system between the gastrointestinal tract and the central nervous system. Gastrointestinal disorders such as irritable bowel syndrome and inflammatory bowel disease have been linked to various psychiatric conditions, including depression, anxiety disorders, and sleep disturbances (Góralczyk‐Bińkowska et al. 2022; Y.‐T. Lee et al. 2015; Gracie et al. 2019; Gracie et al. 2018). It has been hypothesised that IIDs may similarly contribute to the development of psychiatric disorders through disruptions of the gut–brain axis. Supporting this hypothesis, a large‐scale, population‐based cohort study in Taiwan demonstrated that patients with a history of IID had a 2.7‐fold increased risk of developing psychiatric disorders compared with controls, with the risk further elevated in individuals with recurrent infections (Yu et al. 2022).

Nevertheless, longitudinal epidemiological data on the long‐term psychiatric sequelae of IIDs remain scarce, particularly among Asian populations, including those in South Korea. Therefore, the present study aimed to investigate the association between repeated IID diagnoses and the subsequent risk of various psychiatric disorders, including depressive disorder, bipolar disorder, anxiety disorder, obsessive‐compulsive disorder (OCD), adjustment disorder, organic mental disorders, schizophrenia, and alcohol use disorder, using a large, nationally representative cohort from South Korea. Given the paucity of prior research, the study aimed to identify potential associations that may inform future hypothesis‐driven investigations.

## 2 Methods

### 2.1 Data Sources

The dataset used in this study was derived from the National Sample Cohort (NSC), which comprises nationwide insurance claims submitted by healthcare providers between 2002 and 2013. This cohort includes a representative random sample of approximately 1 million individuals, accounting for roughly 2% of the total population enroled in the National Health Insurance Service (NHIS) in 2002. Participants were selected using a stratified random sampling method based on 1476 strata defined by age, sex, and income level (J. Lee et al. 2017).

The Korean NHIS is a mandatory, universal health insurance system that covers approximately 98% of the population and provides routine health screenings every 1–2 years (Shin et al. 2016). The NSC database includes comprehensive information on diagnoses (coded according to the International Classification of Diseases, 10th Revision [ICD‐10]), healthcare utilization, health screening results, and lifestyle factors recorded during the study period.

This study was exempted from ethical review by the Institutional Review Board of Sungshin Women's University (SSWUIRB‐2025‐044). The requirement for informed consent was waived because the NHIS database is anonymised and does not contain identifiable personal information. This study was conducted and reported in accordance with the Strengthening the Reporting of Observational Studies in Epidemiology (STROBE) guidelines (Vandenbroucke et al. 2014).

### 2.2 Participant Selection

The primary exposure in this study was recurrent diagnosis of IID. Independent cohorts were constructed for each of the eight psychiatric outcomes under investigation. Individuals who received three or more diagnoses of IID (ICD‐10 codes A00–A09), recorded as either a primary or secondary diagnosis from 2004 onward, were defined as the case group (Table S1). The index date was defined as the date of the third IID diagnosis. To reduce the potential for reverse causality, a two‐year washout period (2002–2003) was implemented. During this period, individuals with any record of IID or psychiatric disorders were excluded. To minimise confounding, individuals with medical conditions known to be associated with psychiatric morbidity were also excluded. These conditions included congenital malformations of the nervous system, chromosomal abnormalities, neurodegenerative diseases, epilepsy and recurrent seizures, encephalopathy, developmental disorders, intracranial injuries, malignant neoplasms of the brain, and neurosurgical history (Supporting Information S1: Table S2).

Additional exclusion criteria were: (1) absence of health screening data within 2 years prior to the index date; (2) age < 20 or ≥ 80 years at the index date; and (3) any diagnosis of the outcome psychiatric disorders prior to the first IID diagnosis.

**FIGURE 1:**
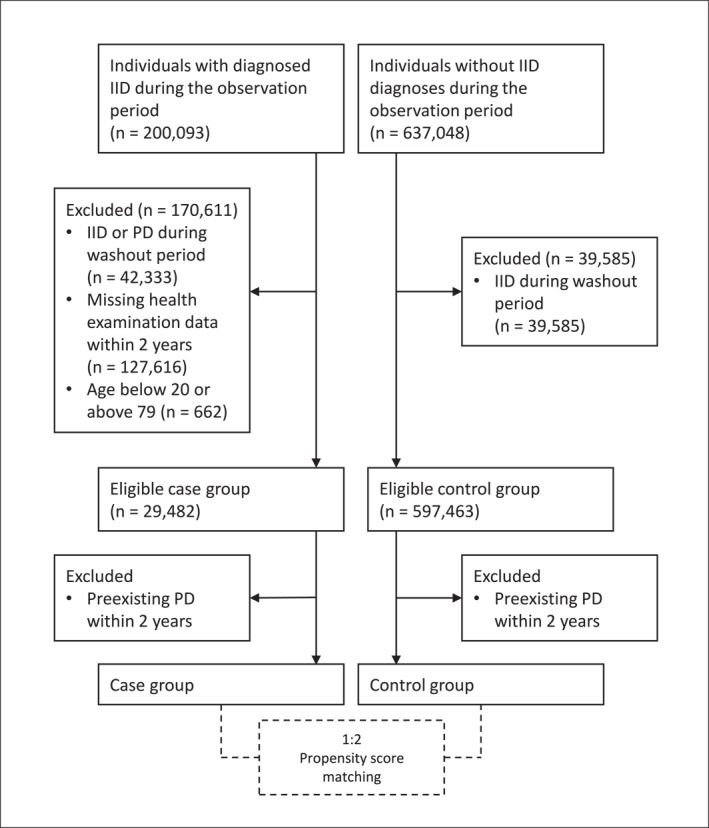
Flowchart of case and control group selection. IID = intestinal infectious disease; PD = psychiatric disease.

The control group comprised individuals with no record of IID diagnosis during the study period and who met the same exclusion criteria applied to the exposed group. Controls were selected at a 1:2 ratio using propensity score matching based on age, sex, and year of health screening. Variables used in propensity score matching included smoking status, alcohol consumption, body mass index (BMI), total cholesterol, systolic blood pressure, diastolic blood pressure, fasting blood sugar, and income level. Missing values were imputed using single imputation with predictive mean matching. The index date for controls was assigned to match the corresponding index date of the cases. The selection process for both cases and controls is illustrated in Figure 1.

To evaluate potential dose‐response relationships, additional cohorts were defined according to the number of IID diagnoses. Separate analyses were conducted for individuals with two, four, five, and six or more documented IID events. The control group was defined as individuals with no recorded diagnosis of IID (ICD‐10 codes A00–A09) throughout the entire study period.

### 2.3 Study Outcome

The primary outcome of interest was the new onset of psychiatric disorders. The psychiatric conditions included depressive disorder (F32, F33), bipolar disorder (F31), anxiety disorder (F40, F41), OCD (F42), adjustment disorder (F43), organic mental disorders (F06), schizophrenia (F20, F21, F22, F23, F25, F28, F29), and alcohol use disorder (F10) (Supporting Information S1: Table S1). The occurrence of each outcome was defined as having at least one medical claim with the corresponding ICD‐10 code recorded in either outpatient or inpatient settings. Although ICD‐10 psychiatric diagnoses can be entered by any physician, in South Korea, such diagnoses are typically made by board‐certified psychiatrists.

### 2.4 Baseline Characteristics

The variables included in the analysis were age, sex, smoking status, alcohol consumption, height, weight, BMI, total cholesterol, systolic blood pressure, diastolic blood pressure, fasting blood glucose, and income level. The data recorded closest to the date of the health examination were used. Height and weight were treated as continuous variables, whereas all other variables were considered categorical.

### 2.5 Statistical Analysis

To compare baseline characteristics between the case and control groups, standardized differences (SD) were calculated, with an SD < 0.1 considered negligible. The crude incidence rate (IR) per 1000 person‐years and IR ratio (IRR) were calculated to compare the occurrence of psychiatric disorders between patients with IID and controls. Cumulative incidence of newly diagnosed psychiatric disorders was estimated using Kaplan–Meier survival analysis, and comparisons between groups were performed using the log‐rank test. Follow‐up ended at the earliest occurrence of a psychiatric disorder diagnosis, death, or the end of the study period (December 31, 2013). Multivariable Cox proportional hazards regression models were used to evaluate the association between IID and each psychiatric disorder, producing adjusted hazard ratios (aHRs) with 95% confidence intervals (CIs). All statistical analyses were performed using R software (version 4.4.1; R Foundation for Statistical Computing, Vienna, Austria), with statistical significance determined using two‐sided tests and a *p*‐value threshold of < 0.05.

## 3 Results

Based on the inclusion and exclusion criteria, separate cohorts of patients with IID were identified for each psychiatric outcome: 27,687 for depressive disorder, 29,411 for bipolar disorder, 26,463 for anxiety disorder, 29,447 for OCD, 29,172 for adjustment disorder, 29,269 for organic mental disorders, 29,394 for schizophrenia, and 29,360 for alcohol use disorder. Person‐years at risk and mean follow‐up durations for each cohort are summarised in Table 1 and Supporting Information S1: Table S3. Standardized differences for baseline characteristics between the IID and control groups were all < 0.1, indicating negligible imbalance across matched cohorts (Supporting Information S1: Table S4).

**TABLE 1:** Crude incidence rates and risk ratios for psychiatric disorders in cases and matched controls.

	Case cohort				Reference cohort				IRR (95% CI)
	No. of Participants	Cases	Person‐years	IR per 1000 person‐years (95% CI)	No. of Participants	Cases	Person‐years	IR per 1000 person‐years (95% CI)	
Depressive disorder	27,687	1754	80,666.87	21.74 (20.73–22.77)	55,374	1701	166,459.46	10.22 (9.74–10.71)	2.13 (1.99–2.27)
Bipolar disorder	29,411	127	89,644.88	1.42 (1.17–1.67)	58,822	139	179,910.71	0.77 (0.64–0.91)	1.83 (1.44–2.33)
Anxiety disorder	26,463	2779	74,422.52	37.34 (35.96–38.74)	52,926	2664	156,632.33	17.01 (16.36–17.66)	2.20 (2.08–2.32)
Obsessive‐compulsive disorder	29,447	48	89,838.46	0.53 (0.39–0.69)	58,894	24	180,358.32	0.13 (0.08–0.19)	4.02 (2.46–6.55)
Adjustment disorder	29,172	450	88,026.27	5.11 (4.65–5.59)	58,344	392	177,809.20	2.20 (1.99–2.42)	2.32 (2.03–2.66)
Organic mental disorders	29,269	454	88,895.14	5.11 (4.65–5.58)	58,538	552	178,923.95	3.09 (2.83–3.35)	1.66 (1.46–1.87)
Schizophrenia	29,394	86	89,632.42	0.96 (0.76–1.17)	58,788	117	179,893.11	0.65 (0.53–0.77)	1.48 (1.12–1.95)
Alcohol use disorder	29,360	104	89,442.36	1.16 (0.95–1.39)	58,720	162	179,484.07	0.90 (0.77–1.04)	1.29 (1.01–1.65)

Abbreviations: IR = incidence rate; IRR = incidence rate ratio.

The IRs and IRRs for each psychiatric disorder are presented in Table 1. Anxiety disorder had the highest IR among the outcomes, with 2779 cases (10.5%) in the IID group and 2664 cases (5.03%) in the control group. The IR per 1000 person‐years was 37.34 (95% CI, 35.96–38.74) and 17.01 (95% CI, 16.36–17.66) in the IID and control groups, respectively. Depressive disorder showed the second highest IR, with 1754 cases (6.3%) in the IID group and 1701 (3.1%) in the control group; the corresponding IRs were 21.74 (95% CI, 20.73–22.77) and 10.22 (95% CI, 9.74–10.71), respectively. In contrast, OCD exhibited the lowest IR, with 0.53 (95% CI, 0.39–0.69) and 0.13 (95% CI, 0.08–0.19) in the IID and control groups, respectively.

All psychiatric outcomes demonstrated significantly elevated IRRs in the IID group compared with the control group. The highest IRR was observed for OCD (4.02; 95% CI, 2.46–6.55), whereas the lowest was for alcohol use disorder (1.29; 95% CI, 1.01–1.65).

Supporting Information S1: Table S5 presents the IRRs stratified by age, sex, smoking status, alcohol consumption, BMI, total cholesterol level, and income. Notably, current smokers exhibited higher IRRs for all psychiatric outcomes except adjustment disorder, compared with never or former smokers. Additionally, individuals who consumed alcohol ≥ 3 times per week showed higher IRRs for all outcomes except alcohol use disorder, compared with non‐drinkers or those drinking 1–2 times per week.

Supporting Information S1: Figure S1 presents the 10‐year cumulative incidence of psychiatric disorders based on Kaplan–Meier survival curves. According to the log‐rank test, the IID group exhibited a significantly higher cumulative incidence of all psychiatric outcomes compared with the control group over the follow‐up period.

**FIGURE 2:**
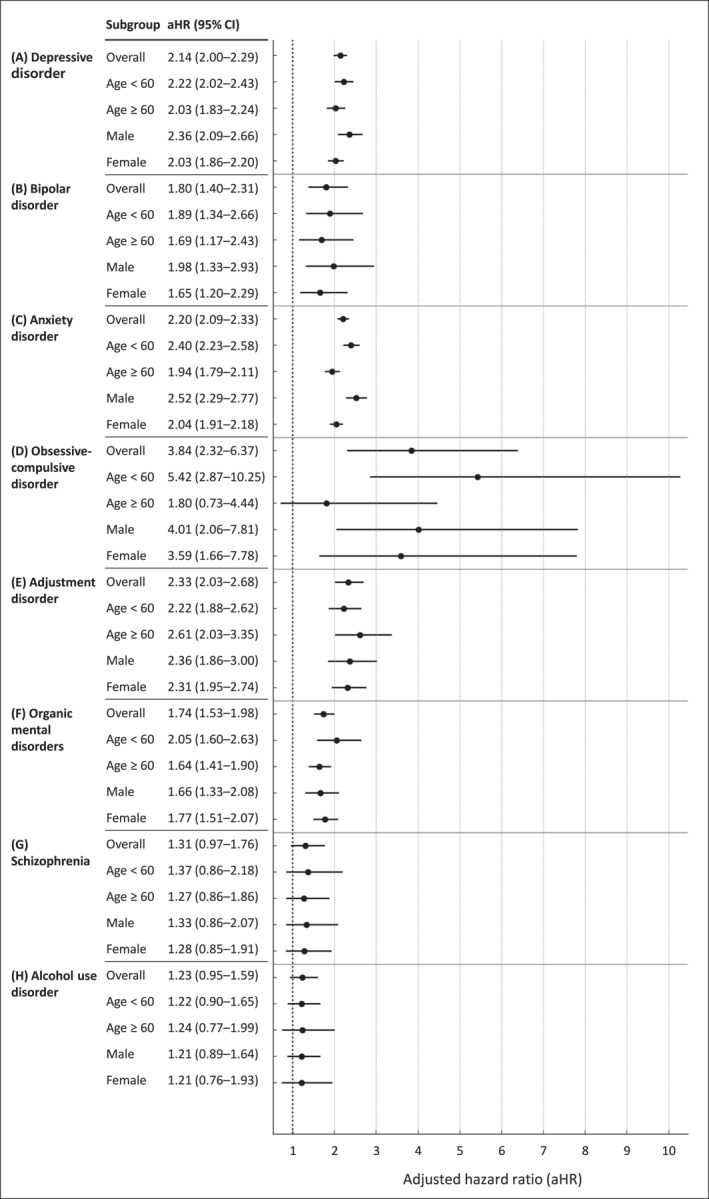
Forest plot of adjusted hazard ratios for psychiatric disorders in patients with three intestinal infectious disease (IID) diagnoses, based on multivariable cox proportional hazards model. Adjusted for age, sex, smoking status, alcohol consumption, body mass index (BMI), total cholesterol, and income.

Multivariable Cox proportional hazards regression analysis showed that patients with IID had a significantly increased risk of six psychiatric disorders, including depressive disorder, bipolar disorder, anxiety disorder, OCD, adjustment disorder, and organic mental disorders (Figure 2). The model was adjusted for age, sex, smoking status, alcohol consumption, BMI, total cholesterol, and income level. Among these, OCD showed the highest aHR at 3.84 (95% CI, 2.32–6.37), followed by adjustment disorder (aHR, 2.33; 95% CI, 2.03–2.68) and anxiety disorder (aHR, 2.20; 95% CI, 2.09–2.33). In contrast, the associations for schizophrenia (aHR, 1.31; 95% CI, 0.97–1.76) and alcohol use disorder (aHR, 1.23; 95% CI, 0.95–1.59) were not statistically significant.

Subgroup analyses revealed age‐ and sex‐specific differences in psychiatric risk (Figure 2). For OCD, aHR was significantly elevated among individuals aged < 60 years (aHR, 5.42; 95% CI, 2.87–10.25), whereas no significant association was observed in those aged ≥ 60 years (aHR, 1.80; 95% CI, 0.73–4.44). In the case of anxiety disorder, the risk was higher in younger individuals (aHR, 2.40; 95% CI, 2.23–2.58) compared with older adults (aHR, 1.94; 95% CI, 1.79–2.11). Notably, statistically significant differences in aHRs between males and females were observed for depressive and anxiety disorders. For depressive disorder, the aHR was higher in males (aHR, 2.36; 95% CI, 2.09–2.66) than in females (aHR, 2.03; 95% CI, 1.86–2.20). Similarly, for anxiety disorder, males showed a higher risk (aHR, 2.52; 95% CI, 2.29–2.77) compared to females (aHR, 2.04; 95% CI, 1.91–2.18).

**FIGURE 3:**
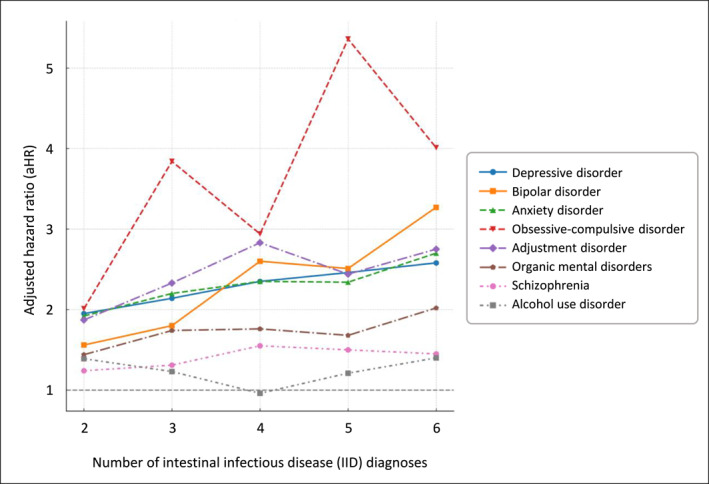
Adjusted hazard ratios for psychiatric disorders according to the number of intestinal infectious disease (IID) episodes.

An additional analysis was conducted to assess the risk of psychiatric disorders based on the number of IID diagnoses. The number of cases and controls, as well as the mean follow‐up duration for each diagnosis frequency category, are presented in Supporting Information S1: Table S3. In multivariable Cox proportional hazards regression, an overall dose‐response relationship was observed, with the risk of major psychiatric disorders increasing progressively with a higher number of IID diagnoses (Figure 3).

For depressive disorder, aHR increased from 1.95 (95% CI, 1.86–2.06) in patients diagnosed twice to 2.58 (95% CI, 2.25–2.95) in those diagnosed six or more times. For bipolar disorder, aHR rose from 1.56 (95% CI, 1.29–1.88) to 3.27 (95% CI, 1.97–5.44); for anxiety disorder, from 1.92 (95% CI, 1.84–2.00) to 2.70 (95% CI, 2.43–3.01); for OCD, from 2.01 (95% CI, 1.40–2.89) to 4.01 (95% CI, 1.37–11.74); for adjustment disorder, from 1.87 (95% CI, 1.69–2.07) to 2.75 (95% CI, 2.06–3.68); and for organic mental disorders, from 1.44 (95% CI, 1.31–1.59) to 2.02 (95% CI, 1.60–2.56).

In contrast, no clear dose‐response relationship was observed for schizophrenia and alcohol use disorder. For schizophrenia, the highest risk was observed among individuals with four IID diagnoses (aHR, 1.55; 95% CI, 1.05–2.28), whereas the risk declined with additional diagnoses and was no longer statistically significant. For alcohol use disorder, the lowest aHR was observed in those with four IID diagnoses (aHR, 0.96; 95% CI, 0.68–1.37), whereas elevated aHRs were noted at two and six or more diagnoses.

## 4 Discussion

### 4.1 Summary of Main Findings

In this nationwide cohort study involving a population representative of the South Korean adults, IID was significantly associated with an increased risk of multiple psychiatric disorders. Individuals with three or more IID diagnoses exhibited markedly elevated risks of developing depressive disorder, bipolar disorder, anxiety disorder, OCD, adjustment disorder, and organic mental disorders. In contrast, no statistically significant associations were identified for schizophrenia or alcohol use disorder. A dose‐response relationship was observed, with a higher frequency of IID diagnoses corresponding to a progressively increased risk of psychiatric morbidity.

### 4.2 Comparison With Previous Literature

These findings are consistent with prior research. A large population‐based cohort study from Taiwan, which followed 150,995 individuals over a 16‐year period, including 30,199 patients with IID and 120,796 matched controls, reported a psychiatric disorder incidence of 13.32% in the IID group and 6.72% in the control group, with an aHR of 2.72 (95% CI, 2.48–2.98) (Yu et al. 2022). This supports the current findings, indicating that IID may represent a significant risk factor for subsequent psychiatric morbidity.

Moreover, studies examining specific pathogens, such as *Toxoplasma gondii*, *Helicobacter pylori*, and *Enterobius vermicularis*, have reported associations with psychiatric outcomes including schizophrenia, bipolar disorder, depression, and anxiety, further supporting the present findings (Sutterland et al. 2015; Li et al. 2024; Chao et al. 2019).

### 4.3 The Gut‐Brain Axis (GBA)

The gut‐brain axis (GBA) is central to understanding the pathophysiological link between IID and psychiatric disorders (Foster and McVey Neufeld 2013; Nikolova et al. 2021). The GBA is a complex bidirectional communication network between the gastrointestinal tract and the central nervous system, mediated through neurological, immunological, and endocrine pathways (Góralczyk‐Bińkowska et al. 2022; Carabotti et al. 2015). Recurrent IID can lead to persistent gut microbiota dysbiosis and impaired intestinal barrier integrity, resulting in metabolic endotoxemia, wherein proinflammatory molecules such as lipopolysaccharides (LPS) continuously enter the systemic circulation.

These systemic inflammatory signals are transmitted to the brain via multiple routes within the GBA. During IID episodes, inflammatory cytokines including interleukin‐1β (IL‐1β), IL‐6, and tumour necrosis factor‐alpha (TNF‐α), released by intestinal epithelial cells, increase the permeability of the blood–brain barrier (BBB), facilitating the influx of these molecules into the central nervous system. This process can induce neuroinflammatory responses characterised by microglial activation and subsequent neuronal and synaptic injury (Loh et al. 2024; Kearns 2024). Neuroinflammation can disrupt synaptic plasticity, suppress the production of brain‐derived neurotrophic factor (BDNF), and interfere with the metabolism of key neurotransmitters such as serotonin and dopamine, ultimately destabilising neural networks and contributing to the pathophysiology of psychiatric disorders (Carabotti et al. 2015; Kouba et al. 2024).

From an endocrine perspective, IID‐related inflammation and vagal nerve activation can lead to chronic stimulation of the hypothalamic–pituitary–adrenal (HPA) axis, resulting in hypercortisolemia and glucocorticoid resistance (Rusch et al. 2023). Dysregulation of the HPA axis is known to play a critical role in the development of several psychiatric conditions, including autism spectrum disorder, anxiety, depression, and schizophrenia (Misiak et al. 2020).

Within the neurological pathway, inflammatory or pathogenic stimuli originating from the gut are transmitted to the nucleus tractus solitarius of the brainstem via afferent vagal fibres. This vagal signalling has been associated with aberrant sensory processing and the manifestation of anxiety‐related behaviours (Goehler et al. 2007). Animal studies have demonstrated that gut bacteria such as *Lactobacillus* and *Bifidobacterium* can modulate neurotransmitter systems, including gamma‐aminobutyric acid (GABA) receptors, through vagal pathways, thereby significantly affecting cognitive and emotional behaviours (Bravo et al. 2011; Bercik et al. 2011; Vazquez et al. 2016).

Collectively, chronic alterations in the gut environment induced by IID may lead to a widespread disruption of the GBA, involving immune, neuroendocrine, and neurotransmission pathways. This complex pathophysiological cascade may impair central nervous system function and, in turn, elevate the risk of developing diverse psychiatric disorders.

The gut microbiota plays a key role in regulating brain biochemistry and behaviour. IID can disrupt the composition and function of the gut microbiota, a condition referred to as gut dysbiosis, which in turn may alter visceral sensory signalling, hypothalamic–pituitary–adrenal axis activity, and the metabolism of neurotransmitters such as serotonin, gamma‐aminobutyric acid, and brain‐derived neurotrophic factor (Carabotti et al. 2015).

Furthermore, systemic inflammatory responses triggered by IID may elevate circulating cytokines, including interleukin‐6 and tumour necrosis factor‐alpha. These cytokines can cross the blood‐brain barrier and influence neuronal function and synaptic plasticity. Such neuroimmune alterations have been implicated in the development of various psychiatric disorders (Foster and McVey Neufeld 2013; Nikolova et al. 2021; Kouba et al. 2024; Goehler et al. 2007; Tzeng et al. 2018).

### 4.4 OCD

Among the psychiatric disorders analysed, OCD exhibited the highest relative risk increase, with an aHR of 3.80. OCD is characterised by intrusive thoughts (obsessions) and repetitive behaviours (compulsions), and has been associated with alterations in the gut microbiota (American Psychiatric Association 2013). Changes in gut microbiome composition have been reported in patients with OCD (Turna et al. 2020; Domènech and et al. 2020). Experimental studies have demonstrated that transplantation of gut microbiota from patients with OCD into gnotobiotic mouse models can induce compulsive‐like behaviours and neuroinflammatory responses (Zhang et al. 2024). Additionally, recurrent intestinal infections may act as chronic stressors that exacerbate obsessive‐compulsive tendencies. Furthermore, anxiety regarding hygiene or cleanliness following infection may manifest as compulsive behaviours (Wahl et al. 2020).

### 4.5 Mood and Anxiety Disorders

The risks of depressive disorder, bipolar disorder, and anxiety disorder was increased by 2.1‐fold, 1.8‐fold, and 2.2‐fold, respectively. For these conditions, as well as for OCD, age‐ and sex‐specific risk patterns were observed. aHRs were higher among younger adults aged < 60 years compared with older adults aged ≥ 60 years, and higher in males than in females.

Younger individuals may experience greater exposure to psychosocial stressors such as academic pressure, employment instability, and interpersonal challenges. Repeated episodes of IID may exacerbate these stress responses, thereby increasing the likelihood of developing depressive and anxiety symptoms (Marwaha et al. 2025). In contrast, among older adults, a relatively stable social support network, greater adaptability to illness, or age‐related changes in immune response to gastrointestinal infection may have contributed to the attenuated psychiatric risk observed in this group.

The higher aHRs reported in males may partly reflect the higher baseline prevalence of psychiatric disorders among females in the control population, potentially diluting the relative risk increase among women (Picco et al. 2017; McLean et al. 2011). In addition, sex‐specific differences in cytokine function (Martin‐Viñas and Quigley 2016), intestinal epithelial barrier permeability (Honda et al. 2016; Pigrau et al. 2016), and gut microbiota composition (Haro et al. 2016) in gastrointestinal diseases may have contributed to the observed sex differences. Further investigation is warranted to elucidate these mechanisms.

### 4.6 Adjustment Disorder

Adjustment disorder is characterised by maladaptive psychological or behavioural responses to identifiable psychosocial stressors. These stressors may include events such as relocation, occupational changes, interpersonal difficulties, medical diagnoses, or accidents (Carta et al. 2009). In the present study, the risk of adjustment disorder was more than two‐fold higher following a diagnosis of IID. This finding suggests that IID may act as a significant emotional stressor that heightens psychological vulnerability.

### 4.7 Organic Mental Disorders

Patients in the IID cohort exhibited a 1.67‐fold higher risk of developing organic mental disorders. These conditions are typically attributed to structural or functional abnormalities of the brain. Chronic inflammation resulting from recurrent IID may influence central nervous system function and contribute to the onset of these conditions. The gut microbiota has been shown to affect neurogenesis, microglial activation, and blood–brain barrier permeability (Tremlett et al. 2017; Erny et al. 2015; Braniste et al. 2014) suggesting a plausible mechanistic link between IID and the pathogenesis of organic mental disorders. The gut microbiota is known to influence neurogenesis, microglial activation, and the permeability of the blood‐brain barrier. Through these mechanisms, IID may plausibly contribute to the pathogenesis of organic mental disorders. These findings suggest that IID, although primarily a gastrointestinal condition, may exert systemic effects, including on neuropsychiatric health. Clinicians should be aware of the potential neuropsychiatric consequences of IID, particularly in individuals at elevated risk for organic mental disorders.

### 4.8 Schizophrenia and Alcohol Use Disorder

Although schizophrenia and alcohol use disorder were not significantly associated with IID in the primary analysis, a statistically significant increase in risk was observed among individuals with a history of two or more, or four or more IID diagnoses. A population‐based cohort study from Taiwan similarly reported significantly elevated risks for schizophrenia (aHR, 3.299) and alcohol use disorder (aHR, 2.224) among individuals with IID (Yu et al. 2022). The discrepancy between the two studies may be attributable to limited statistical power in the present analysis, given the relatively low prevalence of schizophrenia and alcohol use disorder. These findings underscore the need for further investigation using larger cohorts to more precisely characterise the potential association between IID and the risk of schizophrenia and alcohol use disorder.

### 4.9 Psychiatric Risk in Relation to Frequency of IID Diagnosis

Analysis based on the frequency of IID diagnosis suggested that repeated episodes of IID may increase the risk of developing various psychiatric disorders. A dose‐response relationship was observed, wherein the risk of depressive disorder, bipolar disorder, anxiety disorder, OCD, adjustment disorder, and organic mental disorders increased proportionally with the number of IID diagnoses.

Similar findings were reported in a population‐based cohort study from Taiwan, which demonstrated that patients with three or more IID‐related medical visits had a significantly higher risk of psychiatric disorders (aHR, 3.918; 95% CI, 3.569–4.280) compared with those with only one or two diagnoses (aHR, 2.162; 95% CI, 1.964–2.362) (Yu et al. 2022). These results indicate that the association between IID and psychiatric disorders may be strengthened by cumulative exposure, underscoring the need for heightened clinical attention to mental health in patients with recurrent IID.

### 4.10 Impact of Smoking and Alcohol Consumption

In the present study, both smoking and alcohol consumption were associated with an increased risk of psychiatric disorders following IID diagnosis. Smoking is a well‐established risk factor for various psychiatric disorders, including depressive disorder, bipolar disorder, and schizophrenia (Hu 2024). Nicotine, a major component of tobacco, may disrupt neurotransmitter regulation and induce neuroinflammation, thereby negatively affecting mental health (Yuan et al. 2020). In addition, toxicants in cigarette alter gut microbial composition, resulting in gut dysbiosis (Gui et al. 2021). These adverse effects of smoking may act as mediators that strengthen the link between gastrointestinal infections and psychiatric outcomes.

Similarly, alcohol consumption can significantly disrupt the gut‐brain axis (Shukla and Hsu 2025). Ethanol damages intestinal epithelial cells, increases gut permeability, and induces microbial imbalance (Bishehsari et al. 2017; Hillemacher et al. 2018). When these alcohol‐related effects coincide with infectious insults to the gut, the resulting increase in systemic exposure to endotoxins and inflammatory mediators may further exacerbate neuropsychiatric vulnerability.

These findings suggest that individuals with unhealthy alcohol or smoking habits who also experience recurrent IID may be at substantially elevated risk of developing psychiatric disorders. These results emphasise the need for targeted behavioural interventions, including smoking cessation and alcohol avoidance, in patients with IID at increased risk for psychiatric morbidity.

### 4.11 Implications

Although IID is a common clinical condition, its psychological consequences are often underrecognized. The present study highlights the need for integrated mental health screening and early psychiatric intervention in individuals with IID, particularly those with recurrent infections or other risk‐enhancing characteristics. Beyond the immediate management of gastrointestinal symptoms, the prevention and appropriate treatment of IID may serve as a strategy to mitigate long‐term psychiatric morbidity. These findings also underscore the broader significance of basic public health initiatives—such as ensuring access to clean water, improving food safety, and implementing effective infection control measures—not only in reducing the burden of enteric infections but also in promoting mental health at the population level.

### 4.12 Strengths and Limitations

This study leveraged a large‐scale, nationwide cohort representative of the South Korean population, enabling robust longitudinal analysis of the association between recurrent IID and the risk of various psychiatric disorders. The extended follow‐up period and substantial sample size enhanced the statistical power and supported temporal inference of the observed associations.

Nevertheless, several limitations should be acknowledged. First, this study utilised health insurance claims data based on ICD‐10 codes, which may be subject to diagnostic inaccuracies and misclassification. Individuals with mild symptoms who did not seek medical attention may have been excluded, potentially leading to underestimation of IID incidence. Second, detailed clinical information, such as IID severity, causative pathogens, and treatment modalities, was not available, limiting assessment of their potential influence on psychiatric outcomes. Similarly, data on the clinical characteristics or severity of psychiatric disorders were limited. Third, as an epidemiological investigation, this study could not directly examine the biological mechanisms through which IID may contribute to psychiatric disorders, such as alterations in gut microbiota, inflammatory responses, or neurotransmitter dysregulation. Fourth, although propensity score matching was employed to adjust for major confounders, residual confounding from unmeasured psychosocial factors or lifestyle variables cannot be fully ruled out. Fifth, while individuals with major neurodevelopmental and neurological conditions were excluded, the possibility remains that other comorbidities—such as chronic inflammatory or autoimmune diseases—may influence both gastrointestinal and neuropsychiatric function. These unmeasured conditions may have contributed to the observed associations and warrant further investigation. Lastly, given the observational nature of the study design, the potential for reverse causality or protopathic bias cannot be entirely excluded. Although a washout period was applied to reduce the risk of such biases, future studies should consider employing time‐dependent analytical approaches to more accurately delineate the temporal relationship between recurrent IID and the development of psychiatric disorders.

Further investigation is warranted to elucidate the biological pathways linking IID to psychiatric morbidity, particularly those involving changes in the gut microbiome, immune activation, and neurophysiological dysfunction, and evaluate the effectiveness of preventive and interventional strategies in this context.

## 5 Conclusion

This nationwide population‐based cohort study demonstrated a significant association between IID and an increased risk of multiple psychiatric disorders. In particular, recurrent IID was significantly associated with elevated risks of depressive disorder, bipolar disorder, anxiety disorder, OCD, adjustment disorder, and organic mental disorders. A dose‐response relationship was also observed, with an increasing number of IID diagnoses corresponding to a progressively increased risk of psychiatric morbidity. These findings suggest that the prevention and appropriate management of IID may contribute not only to gastrointestinal health but also to improved mental health outcomes from a public health perspective.

## Ethics Statement

This study was approved by Sungshin Women's University (SSWUIRB‐2025‐044).

## Consent

Informed consent was waived due to the use of anonymised data.

## Conflicts of Interest

The authors declare no conflicts of interest.

## Supporting information


Supporting Information S1

Supporting Information S2

## Data Availability

The datasets analysed in this study can be obtained from the corresponding authors upon reasonable request.
