# Supplementary material for: Psychiatric Morbidity Following Intestinal Infectious Diseases: A Nationwide Cohort Study in South Korea
Source: Stress Health. 2025 Aug 29;41(5):e70103. doi: 10.1002/smi.70103 (PMC12397681; doi:10.1002/smi.70103)

**Supplementary Materials**

**Table S1.** International Classification of Diseases, 10th revision (ICD-10) codes for intestinal infectious diseases (IID) and psychiatric disorders.

| **Diseases** | **ICD-10 codes** |
| --- | --- |
| Intestinal infectious disease | A00–09 |
| Psychiatric disorders |  |
| Depressive disorder | F32, F33 |
| Bipolar disorder | F31 |
| Anxiety disorder | F40, F41 |
| Obsessive-compulsive disorder | F42 |
| Adjustment disorder | F43 |
| Organic mental disorders | F06 |
| Schizophrenia | F20, F21, F22, F23, F25, F28, F29 |
| Alcohol use disorder | F10 |

**Table S2.** International Classification of Diseases, 10th Revision (ICD-10) codes for participant exclusion.

| **Diseases** | **ICD-10 Codes** |
| --- | --- |
| Congenital malformations of the nervous system | Q00–Q07 |
| Chromosomal abnormalities | Q90–Q99 |
| Degenerative diseases of the nervous system | G30–G32 |
| Epilepsy and recurrent seizures | G40–G41 |
| Encephalopathy | G93.4 |
| Pervasive developmental disorders | F84 |
| Intracranial injury | S06 |
| History of malignant neoplasm of brain | Z85.841 |
| History of brain surgery | Z90.81 |

**Table S3.** Mean follow-up duration of study participants according to the number of intestinal infectious disease (IID) diagnoses.

| **Number of IID diagnoses** | **Psychiatric outcome** | **Group** | **Number of Participants** | **Mean follow-up (SD, years)** |
| --- | --- | --- | --- | --- |
| 2 | Depressive disorder | Case | 50224 | 3.37 (2.48) |
|  |  | Control | 100448 | 3.46 (2.49) |
|  | Bipolar disorder | Case | 52759 | 3.51 (2.51) |
|  |  | Control | 105518 | 3.52 (2.51) |
|  | Anxiety disorder | Case | 48442 | 3.26 (2.45) |
|  |  | Control | 96884 | 3.40 (2.47) |
|  | Obsessive-compulsive disorder | Case | 52839 | 3.52 (2.51) |
|  |  | Control | 105678 | 3.52 (2.51) |
|  | Adjustment disorder | Case | 52425 | 3.48 (2.50) |
|  |  | Control | 104850 | 3.51 (2.50) |
|  | Organic mental disorders | Case | 52612 | 3.50 (2.51) |
|  |  | Control | 105224 | 3.52 (2.50) |
|  | Schizophrenia | Case | 52742 | 3.52 (2.51) |
|  |  | Control | 105484 | 3.52 (2.51) |
|  | Alcohol use disorder | Case | 52701 | 3.51 (2.51) |
|  |  | Control | 105402 | 3.52 (2.51) |
| 3 | Depressive disorder | Case | 29269 | 2.91 (2.22) |
|  |  | Control | 58538 | 3.01 (2.25) |
|  | Bipolar disorder | Case | 29360 | 3.05 (2.27) |
|  |  | Control | 58720 | 3.06 (2.27) |
|  | Anxiety disorder | Case | 29394 | 2.81 (2.19) |
|  |  | Control | 58788 | 2.96 (2.24) |
|  | Obsessive-compulsive disorder | Case | 29411 | 3.05 (2.27) |
|  |  | Control | 58822 | 3.06 (2.27) |
|  | Adjustment disorder | Case | 27687 | 3.02 (2.26) |
|  |  | Control | 55374 | 3.05 (2.26) |
|  | Organic mental disorders | Case | 26463 | 3.04 (2.26) |
|  |  | Control | 52926 | 3.06 (2.27) |
|  | Schizophrenia | Case | 29447 | 3.05 (2.27) |
|  |  | Control | 58894 | 3.06 (2.27) |
|  | Alcohol use disorder | Case | 29172 | 3.05 (2.27) |
|  |  | Control | 58344 | 3.06 (2.27) |
| 4 | Depressive disorder | Case | 16441 | 2.65 (2.06) |
|  |  | Control | 32882 | 2.74 (2.08) |
|  | Bipolar disorder | Case | 17627 | 2.78 (2.10) |
|  |  | Control | 35254 | 2.79 (2.10) |
|  | Anxiety disorder | Case | 15603 | 2.55 (2.02) |
|  |  | Control | 31206 | 2.70 (2.07) |
|  | Obsessive-compulsive disorder | Case | 17652 | 2.78 (2.10) |
|  |  | Control | 35304 | 2.80 (2.10) |
|  | Adjustment disorder | Case | 17479 | 2.76 (2.10) |
|  |  | Control | 34958 | 2.79 (2.10) |
|  | Organic mental disorders | Case | 17542 | 2.77 (2.10) |
|  |  | Control | 35084 | 2.79 (2.10) |
|  | Schizophrenia | Case | 17628 | 2.78 (2.10) |
|  |  | Control | 35256 | 2.79 (2.10) |
|  | Alcohol use disorder | Case | 17599 | 2.78 (2.10) |
|  |  | Control | 35198 | 2.79 (2.10) |
| 5 | Depressive disorder | Case | 10267 | 2.48 (1.96) |
|  |  | Control | 20534 | 2.57 (1.98) |
|  | Bipolar disorder | Case | 11100 | 2.60 (2.00) |
|  |  | Control | 22200 | 2.62 (2.00) |
|  | Anxiety disorder | Case | 9659 | 2.38 (1.92) |
|  |  | Control | 19318 | 2.53 (1.97) |
|  | Obsessive-compulsive disorder | Case | 11130 | 2.61 (2.00) |
|  |  | Control | 22260 | 2.62 (2.00) |
|  | Adjustment disorder | Case | 10989 | 2.58 (2.00) |
|  |  | Control | 21978 | 2.61 (2.00) |
|  | Organic mental disorders | Case | 11044 | 2.59 (2.00) |
|  |  | Control | 22088 | 2.61 (2.00) |
|  | Schizophrenia | Case | 11111 | 2.61 (2.00) |
|  |  | Control | 22222 | 2.62 (2.00) |
|  | Alcohol use disorder | Case | 11093 | 2.61 (2.01) |
|  |  | Control | 22186 | 2.62 (2.00) |
| 6 | Depressive disorder | Case | 6800 | 2.39 (1.91) |
|  |  | Control | 13600 | 2.48 (1.94) |
|  | Bipolar disorder | Case | 7436 | 2.51 (1.94) |
|  |  | Control | 14872 | 2.52 (1.94) |
|  | Anxiety disorder | Case | 6416 | 2.28 (1.88) |
|  |  | Control | 12832 | 2.44 (1.92) |
|  | Obsessive-compulsive disorder | Case | 7457 | 2.52 (1.94) |
|  |  | Control | 14914 | 2.52 (1.94) |
|  | Adjustment disorder | Case | 7359 | 2.49 (1.94) |
|  |  | Control | 14718 | 2.51 (1.94) |
|  | Organic mental disorders | Case | 7395 | 2.50 (1.94) |
|  |  | Control | 14790 | 2.51 (1.94) |
|  | Schizophrenia | Case | 7442 | 2.52 (1.94) |
|  |  | Control | 14884 | 2.52 (1.94) |
|  | Alcohol use disorder | Case | 7430 | 2.52 (1.95) |
|  |  | Control | 14860 | 2.53 (1.95) |

Abbreviations: IID = intestinal infectious disease; SD = standard deviation.

**Table S4.** Baseline demographic characteristics of patients with intestinal infectious disease (IID) and matched controls.

(A) Depressive disorder

|  | | **Case group**  **(n = 27,687) (%)** | **Control group**  **(n =** **55,374) (%)** | **Standardized difference** |
| --- | --- | --- | --- | --- |
| Age (years) | 20–29 | 3248 (11.7) | 6496 (11.7) | 0.00 |
|  | 30–39 | 4776 (17.2) | 9552 (17.2) |  |
|  | 40–49 | 5954 (21.5) | 11908 (21.5) |  |
|  | 50–59 | 6070 (21.9) | 12140 (21.9) |  |
|  | 60–69 | 4675 (16.9) | 9350 (16.9) |  |
|  | ≥ 70 | 2964 (10.7) | 5928 (10.7) |  |
| Sex | Male | 12893 (46.6) | 25786 (46.6) | 0.00 |
|  | Female | 14794 (53.4) | 29588 (53.4) |  |
| Smoking status | Yes | 4923 (17.8) | 9920 (17.9) | 0.02 |
|  | No | 18503 (66.8) | 36969 (66.8) |  |
|  | Ex-smoking | 3426 (12.4) | 6963 (12.6) |  |
|  | Missing | 835 (3.0) | 1522 (2.7) |  |
| Frequency of alcohol consumption (per week) | 0 | 15318 (55.3) | 30592 (55.3) | 0.00 |
|  | 1–2 | 9029 (32.6) | 18128 (32.7) |  |
|  | ≥ 3 | 2975 (10.7) | 5942 (10.7) |  |
| Weight (kg, mean ± SD) | | 62.64 ± 11.52 | 62.52 ± 11.36 | 0.01 |
| Height (cm, mean ± SD) | | 162.81 ± 9.11 | 162.66 ± 9.15 | 0.02 |
| BMI (kg/m^2^) | < 18.5 | 1312 (4.7) | 2433 (4.4) | 0.02 |
|  | 18.5 to < 25 | 17908 (64.7) | 36234 (65.4) |  |
|  | ≥ 25 | 8459 (30.5) | 16698 (30.2) |  |
| Total cholesterol (mg/dL) | < 200 | 16702 (60.3) | 33273 (60.1) | 0.01 |
|  | ≥ 200 | 10972 (39.6) | 22082 (39.9) |  |
| Systolic blood pressure (mmHg) | < 120 | 11915 (43.0) | 23624 (42.7) | 0.01 |
|  | 120 to < 140 | 12696 (45.9) | 25610 (46.3) |  |
|  | ≥ 140 | 3070 (11.1) | 6135 (11.1) |  |
| Diastolic blood pressure (mmHg) | < 80 | 16142 (58.3) | 32150 (58.0) | 0.01 |
|  | 80 to < 90 | 8925 (32.2) | 17972 (32.5) |  |
|  | ≥ 90 | 2614 (9.4) | 5247 (9.5) |  |
| FBS (mg/dL) | < 100 | 19388 (70.0) | 38765 (70.0) | 0.00 |
|  | 100 to < 126 | 6562 (23.7) | 13096 (23.7) |  |
|  | ≥ 126 | 1729 (6.2) | 3501 (6.3) |  |
| Income | Low | 10315 (37.3) | 20513 (37.0) | 0.01 |
|  | High | 17372 (62.7) | 34861 (63.0) |  |

(B) Bipolar disorder

|  | | **Case group**  **(n = 29,411) (%)** | **Control group**  **(n =** **58,822) (%)** | **Standardized difference** |
| --- | --- | --- | --- | --- |
| Age (years) | 20–29 | 3315 (11.3) | 6630 (11.3) | 0.00 |
|  | 30–39 | 4870 (16.6) | 9740 (16.6) |  |
|  | 40–49 | 6231 (21.2) | 12462 (21.2) |  |
|  | 50–59 | 6482 (22.0) | 12964 (22.0) |  |
|  | 60–69 | 5161 (17.5) | 10322 (17.5) |  |
|  | ≥ 70 | 3352 (11.4) | 6704 (11.4) |  |
| Sex | Male | 13450 (45.7) | 26900 (45.7) | 0.00 |
|  | Female | 15961 (54.3) | 31922 (54.3) |  |
| Smoking status | Yes | 5124 (17.4) | 10326 (17.6) | 0.01 |
|  | No | 19814 (67.4) | 39652 (67.4) |  |
|  | Ex-smoking | 3595 (12.2) | 7209 (12.3) |  |
|  | Missing | 878 (3.0) | 1635 (2.8) |  |
| Frequency of alcohol consumption (per week) | 0 | 16573 (56.3) | 32920 (56.0) | 0.01 |
|  | 1–2 | 9345 (31.8) | 18979 (32.3) |  |
|  | ≥ 3 | 3103 (10.6) | 6127 (10.4) |  |
| Weight (kg, mean ± SD) | | 62.49 ± 11.45 | 62.33 ± 11.31 | 0.01 |
| Height (cm, mean ± SD) | | 162.59 ± 9.11 | 162.42 ± 9.14 | 0.02 |
| BMI (kg/m^2^) | < 18.5 | 1383 (4.7) | 2550 (4.3) | 0.03 |
|  | 18.5 to < 25 | 18999 (64.6) | 38561 (65.6) |  |
|  | ≥ 25 | 9021 (30.7) | 17690 (30.1) |  |
| Total cholesterol (mg/dL) | < 200 | 17680 (60.1) | 35262 (59.9) | 0.00 |
|  | ≥ 200 | 11717 (39.9) | 23535 (40.1) |  |
| Systolic blood pressure (mmHg) | < 120 | 12581 (42.8) | 24986 (42.5) | 0.01 |
|  | 120 to < 140 | 13512 (45.9) | 27217 (46.3) |  |
|  | ≥ 140 | 3312 (11.3) | 6598 (11.2) |  |
| Diastolic blood pressure (mmHg) | < 80 | 17124 (58.3) | 34101 (58.1) | 0.01 |
|  | 80 to < 90 | 9471 (32.2) | 18999 (32.3) |  |
|  | ≥ 90 | 2810 (9.6) | 5702 (9.7) |  |
| FBS (mg/dL) | < 100 | 20525 (69.8) | 41153 (69.9) | 0.01 |
|  | 100 to < 126 | 7008 (23.8) | 13983 (23.8) |  |
|  | ≥ 126 | 1869 (6.4) | 3666 (6.2) |  |
| Income | Low | 10937 (37.2) | 21678 (36.9) | 0.01 |
|  | High | 18474 (62.8) | 37144 (63.1) |  |

(C) Anxiety disorder

|  | | **Case group**  **(n = 26,463) (%)** | **Control group**  **(n = 52,926) (%)** | **Standardized difference** |
| --- | --- | --- | --- | --- |
| Age (years) | 20–29 | 3211 (12.1) | 6422 (12.1) | 0.00 |
|  | 30–39 | 4656 (17.6) | 9312 (17.6) |  |
|  | 40–49 | 5723 (21.6) | 11446 (21.6) |  |
|  | 50–59 | 5732 (21.7) | 11464 (21.7) |  |
|  | 60–69 | 4403 (16.6) | 8806 (16.6) |  |
|  | ≥ 70 | 2738 (10.3) | 5476 (10.3) |  |
| Sex | Male | 12453 (47.1) | 24906 (47.1) | 0.00 |
|  | Female | 14010 (52.9) | 28020 (52.9) |  |
| Smoking status | Yes | 4769 (18.0) | 9704 (18.3) | 0.01 |
|  | No | 17619 (66.6) | 35201 (66.5) |  |
|  | Ex-smoking | 3277 (12.4) | 6502 (12.3) |  |
|  | Missing | 798 (3.0) | 1519 (2.9) |  |
| Frequency of alcohol consumption (per week) | 0 | 14459 (54.6) | 28916 (54.6) | 0.02 |
|  | 1–2 | 8763 (33.1) | 17806 (33.6) |  |
|  | ≥ 3 | 2893 (11.0) | 5483 (10.4) |  |
| Weight (kg, mean ± SD) | | 62.75 ± 11.52 | 62.42 ± 11.16 | 0.03 |
| Height (cm, mean ± SD) | | 162.93 ± 9.08 | 162.79 ± 9.07 | 0.02 |
| BMI (kg/m^2^) | < 18.5 | 1245 (4.7) | 2276 (4.3) | 0.07 |
|  | 18.5 to < 25 | 17067 (64.5) | 35329 (66.8) |  |
|  | ≥ 25 | 8143 (30.8) | 15301 (28.9) |  |
| Total cholesterol (mg/dL) | < 200 | 16033 (60.6) | 32638 (61.7) | 0.03 |
|  | ≥ 200 | 10417 (39.4) | 20266 (38.3) |  |
| Systolic blood pressure (mmHg) | < 120 | 11448 (43.3) | 22760 (43.0) | 0.02 |
|  | 120 to < 140 | 12115 (45.8) | 24512 (46.3) |  |
|  | ≥ 140 | 2895 (11.0) | 5632 (10.6) |  |
| Diastolic blood pressure (mmHg) | < 80 | 15493 (58.6) | 31130 (58.8) | 0.01 |
|  | 80 to < 90 | 8482 (32.1) | 17014 (32.2) |  |
|  | ≥ 90 | 2483 (9.4) | 4764 (9.0) |  |
| FBS (mg/dL) | < 100 | 18566 (70.2) | 38198 (72.2) | 0.07 |
|  | 100 to < 126 | 6221 (23.5) | 11999 (22.7) |  |
|  | ≥ 126 | 1668 (6.3) | 2711 (5.1) |  |
| Income | Low | 9829 (37.1) | 19536 (36.9) | 0.01 |
|  | High | 16634 (62.9) | 33390 (63.1) |  |

(D) Obsessive-compulsive disorder

|  | | **Case group**  **(n = 29,447) (%)** | **Control group**  **(n = 58,894) (%)** | **Standardized difference** |
| --- | --- | --- | --- | --- |
| Age (years) | 20–29 | 3312 (11.2) | 6624 (11.2) | 0.00 |
|  | 30–39 | 4873 (16.5) | 9746 (16.5) |  |
|  | 40–49 | 6235 (21.2) | 12470 (21.2) |  |
|  | 50–59 | 6494 (22.1) | 12988 (22.1) |  |
|  | 60–69 | 5175 (17.6) | 10350 (17.6) |  |
|  | ≥ 70 | 3358 (11.4) | 6716 (11.4) |  |
| Sex | Male | 13457 (45.7) | 26914 (45.7) | 0.00 |
|  | Female | 15990 (54.3) | 31980 (54.3) |  |
| Smoking status | Yes | 5131 (17.4) | 10455 (17.7) | 0.02 |
|  | No | 19841 (67.4) | 39501 (67.0) |  |
|  | Ex-smoking | 3598 (12.2) | 7323 (12.4) |  |
|  | Missing | 877 (3.0) | 1615 (2.7) |  |
| Frequency of alcohol consumption (per week) | 0 | 16600 (56.4) | 32882 (55.6) | 0.02 |
|  | 1–2 | 9348 (31.7) | 18917 (32.1) |  |
|  | ≥ 3 | 3108 (10.6) | 6335 (10.8) |  |
| Weight (kg, mean ± SD) | | 62.49 ± 11.46 | 62.33 ± 11.27 | 0.01 |
| Height (cm, mean ± SD) | | 162.59 ± 9.11 | 162.44 ± 9.17 | 0.02 |
| BMI (kg/m^2^) | < 18.5 | 1383 (4.7) | 2520 (4.3) | 0.03 |
|  | 18.5 to < 25 | 19012 (64.6) | 38695 (65.7) |  |
|  | ≥ 25 | 9044 (30.7) | 17665 (30.0) |  |
| Total cholesterol (mg/dL) | < 200 | 17703 (60.1) | 35666 (60.6) | 0.01 |
|  | ≥ 200 | 11730 (39.9) | 23201 (39.4) |  |
| Systolic blood pressure (mmHg) | < 120 | 12592 (42.8) | 25119 (42.7) | 0.00 |
|  | 120 to < 140 | 13532 (45.9) | 27173 (46.1) |  |
|  | ≥ 140 | 3317 (11.3) | 6592 (11.2) |  |
| Diastolic blood pressure (mmHg) | < 80 | 17143 (58.2) | 34310 (58.3) | 0.01 |
|  | 80 to < 90 | 9484 (32.2) | 19048 (32.3) |  |
|  | ≥ 90 | 2814 (9.6) | 5525 (9.4) |  |
| FBS (mg/dL) | < 100 | 20542 (69.8) | 41277 (70.1) | 0.01 |
|  | 100 to < 126 | 7022 (23.9) | 13929 (23.7) |  |
|  | ≥ 126 | 1874 (6.4) | 3666 (6.2) |  |
| Income | Low | 10958 (37.2) | 21925 (37.2) | 0.00 |
|  | High | 18489 (62.8) | 36969 (62.8) |  |

(E) Adjustment disorder

|  | | **Case group**  **(n = 29,172) (%)** | **Control group**  **(n = 58,344) (%)** | **Standardized difference** |
| --- | --- | --- | --- | --- |
| Age (years) | 20–29 | 3292 (11.3) | 6584 (11.3) | 0.00 |
|  | 30–39 | 4846 (16.6) | 9692 (16.6) |  |
|  | 40–49 | 6188 (21.2) | 12376 (21.2) |  |
|  | 50–59 | 6411 (22.0) | 12822 (22.0) |  |
|  | 60–69 | 5117 (17.5) | 10234 (17.5) |  |
|  | ≥ 70 | 3318 (11.4) | 6636 (11.4) |  |
| Sex | Male | 13368 (45.8) | 26736 (45.8) | 0.00 |
|  | Female | 15804 (54.2) | 31608 (54.2) |  |
| Smoking status | Yes | 5098 (17.5) | 10245 (17.6) | 0.02 |
|  | No | 19632 (67.3) | 39225 (67.2) |  |
|  | Ex-smoking | 3573 (12.2) | 7287 (12.5) |  |
|  | Missing | 869 (3.0) | 1587 (2.7) |  |
| Frequency of alcohol consumption (per week) | 0 | 16420 (56.2) | 32591 (55.7) | 0.01 |
|  | 1–2 | 9290 (31.8) | 18831 (32.3) |  |
|  | ≥ 3 | 3074 (10.5) | 6139 (10.5) |  |
| Weight (kg, mean ± SD) | | 62.51 ± 11.46 | 62.38 ± 11.31 | 0.01 |
| Height (cm, mean ± SD) | | 162.61 ± 9.11 | 162.47 ± 9.14 | 0.02 |
| BMI (kg/m^2^) | < 18.5 | 1373 (4.7) | 2518 (4.3) | 0.03 |
|  | 18.5 to < 25 | 18836 (64.6) | 38285 (65.6) |  |
|  | ≥ 25 | 8955 (30.7) | 17525 (30.0) |  |
| Total cholesterol (mg/dL) | < 200 | 17543 (60.1) | 35252 (60.4) | 0.01 |
|  | ≥ 200 | 11616 (39.9) | 23070 (39.6) |  |
| Systolic blood pressure (mmHg) | < 120 | 12496 (42.8) | 24640 (42.4) | 0.02 |
|  | 120 to < 140 | 13394 (45.9) | 27133 (46.5) |  |
|  | ≥ 140 | 3276 (11.2) | 6555 (11.2) |  |
| Diastolic blood pressure (mmHg) | < 80 | 16994 (58.3) | 33811 (58.1) | 0.01 |
|  | 80 to < 90 | 9389 (32.2) | 18998 (32.6) |  |
|  | ≥ 90 | 2783 (9.5) | 5519 (9.5) |  |
| FBS (mg/dL) | < 100 | 20356 (69.8) | 40759 (69.9) | 0.00 |
|  | 100 to < 126 | 6952 (23.8) | 13897 (23.8) |  |
|  | ≥ 126 | 1856 (6.4) | 3677 (6.3) |  |
| Income | Low | 10860 (37.2) | 21719 (37.2) | 0.00 |
|  | High | 18312 (62.8) | 36625 (62.8) |  |

(F) Organic mental disorders

|  | | **Case group**  **(n = 29,269) (%)** | **Control group**  **(n = 58,538) (%)** | **Standardized difference** |
| --- | --- | --- | --- | --- |
| Age (years) | 20–29 | 3315 (11.3) | 6630 (11.3) | 0.00 |
|  | 30–39 | 4876 (16.7) | 9752 (16.7) |  |
|  | 40–49 | 6223 (21.3) | 12446 (21.3) |  |
|  | 50–59 | 6463 (22.1) | 12926 (22.1) |  |
|  | 60–69 | 5120 (17.5) | 10240 (17.5) |  |
|  | ≥ 70 | 3272 (11.2) | 6544 (11.2) |  |
| Sex | Male | 13402 (45.8) | 26804 (45.8) | 0.00 |
|  | Female | 15867 (54.2) | 31734 (54.2) |  |
| Smoking status | Yes | 5123 (17.5) | 10392 (17.8) | 0.01 |
|  | No | 19701 (67.3) | 39454 (67.4) |  |
|  | Ex-smoking | 3572 (12.2) | 7056 (12.1) |  |
|  | Missing | 873 (3.0) | 1636 (2.8) |  |
| Frequency of alcohol consumption (per week) | 0 | 16459 (56.2) | 32765 (56.0) | 0.01 |
|  | 1–2 | 9327 (31.9) | 18787 (32.1) |  |
|  | ≥ 3 | 3093 (10.6) | 6212 (10.6) |  |
| Weight (kg, mean ± SD) | | 62.51 ± 11.46 | 62.35 ± 11.28 | 0.01 |
| Height (cm, mean ± SD) | | 162.62 ± 9.11 | 162.46 ± 9.17 | 0.02 |
| BMI (kg/m^2^) | < 18.5 | 1377 (4.7) | 2501 (4.3) | 0.03 |
|  | 18.5 to < 25 | 18917 (64.6) | 38333 (65.5) |  |
|  | ≥ 25 | 8967 (30.6) | 17685 (30.2) |  |
| Total cholesterol (mg/dL) | < 200 | 17606 (60.2) | 35497 (60.7) | 0.01 |
|  | ≥ 200 | 11649 (39.8) | 23019 (39.3) |  |
| Systolic blood pressure (mmHg) | < 120 | 12545 (42.9) | 24935 (42.6) | 0.01 |
|  | 120 to < 140 | 13432 (45.9) | 27099 (46.3) |  |
|  | ≥ 140 | 3286 (11.2) | 6491 (11.1) |  |
| Diastolic blood pressure (mmHg) | < 80 | 17055 (58.3) | 33998 (58.1) | 0.01 |
|  | 80 to < 90 | 9415 (32.2) | 18966 (32.4) |  |
|  | ≥ 90 | 2793 (9.5) | 5562 (9.5) |  |
| FBS (mg/dL) | < 100 | 20435 (69.8) | 41037 (70.1) | 0.01 |
|  | 100 to < 126 | 6973 (23.8) | 13805 (23.6) |  |
|  | ≥ 126 | 1852 (6.3) | 3681 (6.3) |  |
| Income | Low | 10895 (37.2) | 21666 (37.0) | 0.01 |
|  | High | 18374 (62.8) | 36872 (63.0) |  |

(G) Schizophrenia

|  | | **Case group**  **(n = 29,394) (%)** | **Control group**  **(n = 58,788) (%)** | **Standardized difference** |
| --- | --- | --- | --- | --- |
| Age (years) | 20–29 | 3312 (11.3) | 6624 (11.3) | 0.00 |
|  | 30–39 | 4871 (16.6) | 9742 (16.6) |  |
|  | 40–49 | 6216 (21.1) | 12432 (21.1) |  |
|  | 50–59 | 6485 (22.1) | 12970 (22.1) |  |
|  | 60–69 | 5158 (17.5) | 10316 (17.6) |  |
|  | ≥ 70 | 3352 (11.4) | 6704 (11.4) |  |
| Sex | Male | 13437 (45.7) | 26874 (45.7) | 0.00 |
|  | Female | 15957 (54.3) | 31914 (54.3) |  |
| Smoking status | Yes | 5128 (17.4) | 10312 (17.5) | 0.00 |
|  | No | 19803 (67.4) | 39642 (67.4) |  |
|  | Ex-smoking | 3590 (12.2) | 7219 (12.3) |  |
|  | Missing | 873 (3.0) | 1615 (2.7) |  |
| Frequency of alcohol consumption (per week) | 0 | 16558 (56.3) | 32908 (56.0) | 0.01 |
|  | 1–2 | 9340 (31.8) | 18844 (32.0) |  |
|  | ≥ 3 | 3106 (10.6) | 6249 (10.6) |  |
| Weight (kg, mean ± SD) | | 62.49 ± 11.46 | 62.39 ± 11.33 | 0.01 |
| Height (cm, mean ± SD) | | 162.59 ± 9.11 | 162.45 ± 9.16 | 0.02 |
| BMI (kg/m^2^) | < 18.5 | 1383 (4.7) | 2548 (4.3) | 0.03 |
|  | 18.5 to < 25 | 18991 (64.6) | 38453 (65.4) |  |
|  | ≥ 25 | 9012 (30.7) | 17774 (30.2) |  |
| Total cholesterol (mg/dL) | < 200 | 17671 (60.1) | 35597 (60.6) | 0.01 |
|  | ≥ 200 | 11709 (39.9) | 23163 (39.4) |  |
| Systolic blood pressure (mmHg) | < 120 | 12575 (42.8) | 24863 (42.3) | 0.01 |
|  | 120 to < 140 | 13501 (45.9) | 27285 (46.4) |  |
|  | ≥ 140 | 3312 (11.3) | 6623 (11.3) |  |
| Diastolic blood pressure (mmHg) | < 80 | 17113 (58.2) | 34098 (58.0) | 0.01 |
|  | 80 to < 90 | 9465 (32.2) | 18958 (32.3) |  |
|  | ≥ 90 | 2810 (9.6) | 5716 (9.7) |  |
| FBS (mg/dL) | < 100 | 20513 (69.8) | 41116 (69.9) | 0.01 |
|  | 100 to < 126 | 7003 (23.8) | 13880 (23.6) |  |
|  | ≥ 126 | 1869 (6.4) | 3776 (6.4) |  |
| Income | Low | 10933 (37.2) | 21851 (37.2) | 0.00 |
|  | High | 18461 (62.8) | 36937 (62.8) |  |

(H) Alcohol use disorder

|  | | **Case group**  **(n = 29,360) (%)** | **Control group**  **(n = 58,720) (%)** | **Standardized difference** |
| --- | --- | --- | --- | --- |
| Age (years) | 20–29 | 3312 (11.3) | 6624 (11.3) | 0.00 |
|  | 30–39 | 4868 (16.6) | 9736 (16.6) |  |
|  | 40–49 | 6206 (21.1) | 12412 (21.1) |  |
|  | 50–59 | 6468 (22.0) | 12936 (22.0) |  |
|  | 60–69 | 5151 (17.5) | 10302 (17.5) |  |
|  | ≥ 70 | 3355 (11.4) | 6710 (11.4) |  |
| Sex | Male | 13378 (45.6) | 26756 (45.6) | 0.00 |
|  | Female | 15982 (54.4) | 31964 (54.4) |  |
| Smoking status | Yes | 5087 (17.3) | 10203 (17.4) | 0.00 |
|  | No | 19820 (67.5) | 39714 (67.6) |  |
|  | Ex-smoking | 3578 (12.2) | 7138 (12.2) |  |
|  | Missing | 875 (3.0) | 1665 (2.8) |  |
| Frequency of alcohol consumption (per week) | 0 | 16573 (56.4) | 32966 (56.1) | 0.01 |
|  | 1–2 | 9320 (31.7) | 18772 (31.9) |  |
|  | ≥ 3 | 3076 (10.6) | 6237 (10.6) |  |
| Weight (kg, mean ± SD) | | 62.48 ± 11.46 | 62.32 ± 11.26 | 0.01 |
| Height (cm, mean ± SD) | | 162.57 ± 9.12 | 162.43 ± 9.17 | 0.02 |
| BMI (kg/m^2^) | < 18.5 | 1379 (4.7) | 2551 (4.3) | 0.03 |
|  | 18.5 to < 25 | 18959 (64.6) | 38493 (65.6) |  |
|  | ≥ 25 | 9014 (30.7) | 17660 (30.1) |  |
| Total cholesterol (mg/dL) | <200 | 17643 (60.1) | 35436 (60.4) | 0.01 |
|  | ≥ 200 | 11703 (39.9) | 23250 (39.6) |  |
| Systolic blood pressure (mmHg) | < 120 | 12567 (42.8) | 24906 (42.4) | 0.01 |
|  | 120 to < 140 | 13486 (45.9) | 27250 (46.4) |  |
|  | ≥ 140 | 3301 (11.2) | 6549 (11.2) |  |
| Diastolic blood pressure (mmHg) | < 80 | 17109 (58.3) | 34097 (58.1) | 0.01 |
|  | 80 to < 90 | 9448 (32.2) | 19038 (32.4) |  |
|  | ≥ 90 | 2797 (9.5) | 5569 (9.5) |  |
| FBS (mg/dL) | <100 | 20501 (69.8) | 41089 (69.9) | 0.01 |
|  | 100 to < 126 | 6993 (23.8) | 13944 (23.7) |  |
|  | ≥ 126 | 1857 (6.3) | 3665 (6.2) |  |
| Income | Low | 10921 (37.2) | 21691 (36.9) | 0.01 |
|  | High | 18439 (62.8) | 37029 (63.1) |  |

Abbreviations: BMI = body mass index; FBS = fasting blood sugar.

**Table S5.** Crude incidence rates and risk ratios for psychiatric disorders among patients with three intestinal infectious disease (IID) diagnoses.

(A) Depressive disorder

|  | | **Case cohort**  **(n = 27,687)** | | | **Reference cohort**  **(n = 55,374)** | | | **IRR**  **(95% CI)** |
| --- | --- | --- | --- | --- | --- | --- | --- | --- |
|  |  | **Cases** | **Person-years** | **IR per 1000 person-years (95% CI)** | **Cases** | **Person-years** | **IR per 1000 person-years (95% CI)** |  |
| Age (years) | < 60 | 1775 | 130990.32 | 13.55 (12.92–14.18) | 1816 | 267226.08 | 6.80 (6.49–7.11) | 1.99 (1.87–2.13) |
|  | ≥ 60 | 1293 | 38453.99 | 33.62 (31.80–35.47) | 1434 | 80454.48 | 17.82 (16.90–18.76) | 1.89 (1.75–2.03) |
| Sex | Male | 1150 | 85234.90 | 13.49 (12.72–14.28) | 1155 | 173929.92 | 6.64 (6.26–7.03) | 2.03 (1.87–2.20) |
|  | Female | 1918 | 84209.41 | 22.78 (21.77–23.80) | 2095 | 173750.65 | 12.06 (11.55–12.58) | 1.89 (1.78–2.01) |
| Sex & Age (years) | Male, < 60 | 693 | 67669.31 | 10.24 (9.49–11.01) | 637 | 137472.05 | 4.63 (4.28–5.00) | 2.21 (1.98–2.46) |
|  | Male, ≥ 60 | 457 | 17565.59 | 26.02 (23.68–28.41) | 518 | 36457.87 | 14.21 (13.00–15.44) | 1.83 (1.61–2.08) |
|  | Female, < 60 | 1082 | 63321.01 | 17.09 (16.08–18.11) | 1179 | 129754.04 | 9.09 (8.57–9.61) | 1.88 (1.73–2.04) |
|  | Female, ≥ 60 | 836 | 20888.40 | 40.02 (37.34–42.75) | 916 | 43996.61 | 20.82 (19.48–22.18) | 1.92 (1.75–2.11) |
| Smoking status | Yes | 460 | 33683.12 | 13.66 (12.41–14.93) | 420 | 69737.00 | 6.02 (5.45–6.61) | 2.27 (1.99–2.59) |
|  | No | 2224 | 111070.46 | 20.02 (19.20–20.86) | 2421 | 228182.87 | 10.61 (10.19–11.04) | 1.89 (1.78–2.00) |
|  | Ex-Smoking | 229 | 14453.69 | 15.84 (13.84–17.92) | 252 | 29532.20 | 8.53 (7.48–9.62) | 1.86 (1.55–2.22) |
| Frequency of alcohol consumption (per week) | 0 | 1984 | 87971.19 | 22.55 (21.56–23.55) | 2130 | 181642.41 | 11.73 (11.23–12.23) | 1.92 (1.81–2.04) |
|  | 1–2 | 744 | 61586.98 | 12.08 (11.22–12.96) | 776 | 124218.93 | 6.25 (5.81–6.69) | 1.93 (1.75–2.14) |
|  | ≥ 3 | 269 | 16270.36 | 16.53 (14.57–18.56) | 270 | 34052.01 | 7.93 (6.99–8.90) | 2.09 (1.76–2.47) |
| BMI (kg/m^2^) | < 18.5 | 138 | 7696.46 | 17.93 (14.94–20.92) | 115 | 15590.87 | 7.38 (6.03–8.72) | 2.43 (1.90–3.11) |
|  | 18.5 to < 25 | 1979 | 110669.21 | 17.88 (17.10–18.68) | 2149 | 228217.01 | 9.42 (9.02–9.82) | 1.90 (1.79–2.02) |
|  | ≥ 25 | 951 | 51012.76 | 18.64 (17.47–19.84) | 986 | 103746.07 | 9.50 (8.92–10.10) | 1.96 (1.79–2.14) |
| Total cholesterol (mg/dL) | < 200 | 1747 | 103141.64 | 16.94 (16.15–17.73) | 1862 | 211318.37 | 8.81 (8.41–9.21) | 1.92 (1.80–2.05) |
|  | ≥ 200 | 1318 | 66139.46 | 19.93 (18.85–21.02) | 1385 | 136000.65 | 10.18 (9.65–10.72) | 1.96 (1.81–2.11) |
| Income | Low | 1106 | 62832.60 | 17.60 (16.57–18.65) | 1230 | 129249.22 | 9.52 (8.99–10.05) | 1.85 (1.71–2.01) |
|  | High | 1962 | 106611.71 | 18.40 (17.60–19.22) | 2020 | 218431.34 | 9.25 (8.84–9.66) | 1.99 (1.87–2.12) |

(B) Bipolar disorder

|  | | **Case cohort**  **(n = 29,411)** | | | **Reference cohort**  **(n = 58,822)** | | | **IRR**  **(95% CI)** |
| --- | --- | --- | --- | --- | --- | --- | --- | --- |
|  |  | **Cases** | **Person-years** | **IR per 1000 person-years (95% CI)** | **Cases** | **Person-years** | **IR per 1000 person-years (95% CI)** |  |
| Age (years) | < 60 | 100 | 140318.85 | 0.71 (0.58–0.86) | 129 | 281177.04 | 0.46 (0.38–0.54) | 1.55 (1.20–2.02) |
|  | ≥ 60 | 107 | 45110.95 | 2.37 (1.93–2.84) | 129 | 90652.33 | 1.42 (1.18–1.68) | 1.67 (1.29–2.15) |
| Sex | Male | 95 | 90629.25 | 1.05 (0.84–1.27) | 112 | 181787.24 | 0.62 (0.51–0.73) | 1.70 (1.29–2.24) |
|  | Female | 112 | 94800.55 | 1.18 (0.97–1.40) | 146 | 190042.13 | 0.77 (0.65–0.89) | 1.54 (1.20–1.97) |
| Sex & Age (years) | Male, < 60 | 46 | 70895.71 | 0.65 (0.47–0.85) | 54 | 142162.95 | 0.38 (0.28–0.49) | 1.71 (1.15–2.53) |
|  | Male, ≥ 60 | 49 | 19733.54 | 2.48 (1.82–3.19) | 58 | 39624.29 | 1.46 (1.11–1.84) | 1.70 (1.16–2.48) |
|  | Female, < 60 | 54 | 69423.14 | 0.78 (0.58–0.99) | 75 | 139014.09 | 0.54 (0.42–0.66) | 1.44 (1.02–2.05) |
|  | Female, ≥ 60 | 58 | 25377.41 | 2.29 (1.73–2.88) | 71 | 51028.04 | 1.39 (1.08–1.72) | 1.64 (1.16–2.32) |
| Smoking status | Yes | 39 | 35658.86 | 1.09 (0.76–1.46) | 41 | 72419.08 | 0.57 (0.40–0.75) | 1.93 (1.25–2.99) |
|  | No | 138 | 123145.90 | 1.12 (0.93–1.31) | 188 | 247760.77 | 0.76 (0.65–0.87) | 1.48 (1.19–1.84) |
|  | Ex-Smoking | 14 | 15467.16 | 0.91 (0.45–1.42) | 18 | 30855.18 | 0.58 (0.32–0.88) | 1.55 (0.77–3.12) |
| Frequency of alcohol consumption (per week) | 0 | 141 | 98871.83 | 1.43 (1.19–1.67) | 182 | 198380.48 | 0.92 (0.79–1.05) | 1.55 (1.25–1.94) |
|  | 1–2 | 39 | 65160.10 | 0.60 (0.41–0.80) | 47 | 129805.30 | 0.36 (0.26–0.47) | 1.65 (1.08–2.53) |
|  | ≥ 3 | 22 | 17316.97 | 1.27 (0.75–1.85) | 21 | 35218.82 | 0.60 (0.37–0.85) | 2.13 (1.17–3.87) |
| BMI (kg/m^2^) | < 18.5 | 9 | 8338.42 | 1.08 (0.48–1.80) | 9 | 16102.26 | 0.56 (0.25–0.93) | 1.93 (0.77–4.86) |
|  | 18.5 to < 25 | 128 | 121162.16 | 1.06 (0.87–1.25) | 155 | 247147.02 | 0.63 (0.53–0.73) | 1.68 (1.33–2.13) |
|  | ≥ 25 | 70 | 55863.33 | 1.25 (0.97–1.56) | 94 | 108483.43 | 0.87 (0.69–1.04) | 1.45 (1.06–1.97) |
| Total cholesterol (mg/dL) | < 200 | 118 | 111981.79 | 1.05 (0.87–1.25) | 148 | 226466.89 | 0.65 (0.55–0.76) | 1.61 (1.27–2.05) |
|  | ≥ 200 | 89 | 73267.88 | 1.21 (0.97–1.47) | 110 | 144969.08 | 0.76 (0.62–0.90) | 1.60 (1.21–2.12) |
| Income | Low | 75 | 68556.92 | 1.09 (0.86–1.34) | 98 | 137678.85 | 0.71 (0.57–0.86) | 1.54 (1.14–2.08) |
|  | High | 132 | 116872.88 | 1.13 (0.94–1.33) | 160 | 234150.52 | 0.68 (0.58–0.79) | 1.65 (1.31–2.08) |

(C) Anxiety disorder

|  | | **Case cohort**  **(n = 26,463)** | | | **Reference cohort**  **(n = 52,926)** | | | **IRR**  **(95% CI)** |
| --- | --- | --- | --- | --- | --- | --- | --- | --- |
|  |  | **Cases** | **Person-years** | **IR per 1000 person-years (95% CI)** | **Cases** | **Person-years** | **IR per 1000 person-years (95% CI)** |  |
| Age (years) | < 60 | 3052 | 123374.80 | 24.74 (23.86–25.62) | 3160 | 255685.88 | 12.36 (11.93–12.79) | 2.00 (1.90–2.10) |
|  | ≥ 60 | 1896 | 34612.67 | 54.78 (52.32–57.26) | 2251 | 73498.49 | 30.63 (29.36–31.89) | 1.79 (1.68–1.90) |
| Sex | Male | 1958 | 80654.76 | 24.28 (23.21–25.35) | 1958 | 167148.95 | 11.71 (11.20–12.23) | 2.07 (1.95–2.21) |
|  | Female | 2990 | 77332.71 | 38.66 (37.28–40.06) | 3453 | 162035.43 | 21.31 (20.60–22.03) | 1.81 (1.73–1.91) |
| Sex & Age (years) | Male, < 60 | 1245 | 64394.91 | 19.33 (18.26–20.42) | 1146 | 132924.32 | 8.62 (8.12–9.13) | 2.24 (2.07–2.43) |
|  | Male, ≥ 60 | 713 | 16259.85 | 43.85 (40.65–47.11) | 812 | 34224.63 | 23.73 (22.12–25.36) | 1.85 (1.67–2.04) |
|  | Female, < 60 | 1807 | 58979.89 | 30.64 (29.23–32.06) | 2014 | 122761.57 | 16.41 (15.70–17.12) | 1.87 (1.75–1.99) |
|  | Female, ≥ 60 | 1183 | 18352.82 | 64.46 (60.81–68.16) | 1439 | 39273.86 | 36.64 (34.76–38.55) | 1.76 (1.63–1.90) |
| Smoking status | Yes | 730 | 32004.84 | 22.81 (21.18–24.47) | 667 | 66979.95 | 9.96 (9.21–10.72) | 2.29 (2.06–2.54) |
|  | No | 3594 | 102739.13 | 34.98 (33.84–36.13) | 4074 | 214007.19 | 19.04 (18.45–19.63) | 1.84 (1.76–1.92) |
|  | Ex-Smoking | 359 | 13726.00 | 26.15 (23.46–28.92) | 404 | 28835.25 | 14.01 (12.66–15.40) | 1.87 (1.62–2.15) |
| Frequency of alcohol consumption (per week) | 0 | 3139 | 80955.57 | 38.77 (37.43–40.13) | 3499 | 170504.65 | 20.52 (19.85–21.20) | 1.89 (1.80–1.98) |
|  | 1–2 | 1285 | 58222.81 | 22.07 (20.87–23.29) | 1335 | 118821.07 | 11.24 (10.64–11.84) | 1.96 (1.82–2.12) |
|  | ≥ 3 | 418 | 15445.95 | 27.06 (24.47–29.72) | 442 | 32582.62 | 13.57 (12.31–14.85) | 1.99 (1.75–2.28) |
| BMI (kg/m^2^) | < 18.5 | 182 | 7317.25 | 24.87 (21.32–28.56) | 191 | 14867.02 | 12.85 (11.03–14.73) | 1.94 (1.58–2.37) |
|  | 18.5 to < 25 | 3221 | 102839.25 | 31.32 (30.24–32.41) | 3642 | 215757.46 | 16.88 (16.33–17.43) | 1.86 (1.77–1.95) |
|  | ≥ 25 | 1543 | 47771.56 | 32.30 (30.69–33.91) | 1573 | 98453.99 | 15.98 (15.19–16.77) | 2.02 (1.88–2.17) |
| Total cholesterol (mg/dL) | < 200 | 2835 | 96534.79 | 29.37 (28.29–30.46) | 3067 | 201942.32 | 15.19 (14.65–15.73) | 1.93 (1.84–2.03) |
|  | ≥ 200 | 2105 | 61311.97 | 34.33 (32.88–35.80) | 2340 | 126929.05 | 18.44 (17.69–19.18) | 1.86 (1.76–1.98) |
| Income | Low | 1870 | 58246.73 | 32.10 (30.66–33.56) | 1975 | 123324.26 | 16.01 (15.31–16.73) | 2.00 (1.88–2.14) |
|  | High | 3078 | 99740.73 | 30.86 (29.78–31.95) | 3436 | 205860.11 | 16.69 (16.14–17.25) | 1.85 (1.76–1.94) |

(D) Obsessive-compulsive disorder

|  | | **Case cohort**  **(n = 29,447)** | | | **Reference cohort**  **(n = 58,894)** | | | **IRR**  **(95% CI)** |
| --- | --- | --- | --- | --- | --- | --- | --- | --- |
|  |  | **Cases** | **Person-years** | **IR per 1000 person-years (95% CI)** | **Cases** | **Person-years** | **IR per 1000 person-years (95% CI)** |  |
| Age (years) | < 60 | 48 | 140459.76 | 0.34 (0.25–0.44) | 45 | 281456.08 | 0.16 (0.11–0.21) | 2.14 (1.42–3.21) |
|  | ≥ 60 | 16 | 45401.45 | 0.35 (0.20–0.53) | 14 | 91031.49 | 0.15 (0.08–0.24) | 2.29 (1.12–4.69) |
| Sex | Male | 40 | 90752.94 | 0.44 (0.31–0.58) | 30 | 182026.91 | 0.16 (0.11–0.23) | 2.67 (1.67–4.29) |
|  | Female | 24 | 95108.27 | 0.25 (0.16–0.36) | 29 | 190460.65 | 0.15 (0.10–0.21) | 1.66 (0.96–2.85) |
| Sex & Age (years) | Male, < 60 | 29 | 70918.47 | 0.41 (0.27–0.56) | 24 | 142265.20 | 0.17 (0.11–0.24) | 2.42 (1.41–4.16) |
|  | Male, ≥ 60 | 11 | 19834.48 | 0.55 (0.25–0.91) | 6 | 39761.70 | 0.15 (0.05–0.28) | 3.68 (1.36–9.94) |
|  | Female, < 60 | 19 | 69541.29 | 0.27 (0.16–0.40) | 21 | 139190.87 | 0.15 (0.09–0.22) | 1.81 (0.97–3.37) |
|  | Female, ≥ 60 | 5 | 25566.98 | 0.20 (0.04–0.39) | 8 | 51269.78 | 0.16 (0.06–0.27) | 1.25 (0.41–3.83) |
| Smoking status | Yes | 14 | 35701.87 | 0.39 (0.20–0.62) | 8 | 71976.67 | 0.11 (0.04–0.19) | 3.53 (1.48–8.41) |
|  | No | 40 | 123493.80 | 0.32 (0.23–0.43) | 42 | 248151.43 | 0.17 (0.12–0.22) | 1.91 (1.24–2.95) |
|  | Ex-Smoking | 6 | 15491.96 | 0.39 (0.13–0.71) | 8 | 30910.95 | 0.26 (0.10–0.45) | 1.50 (0.52–4.31) |
| Frequency of alcohol consumption (per week) | 0 | 31 | 99265.10 | 0.31 (0.21–0.42) | 35 | 197824.30 | 0.18 (0.12–0.24) | 1.77 (1.09–2.86) |
|  | 1–2 | 25 | 65156.01 | 0.38 (0.25–0.54) | 23 | 131273.72 | 0.18 (0.11–0.25) | 2.19 (1.24–3.86) |
|  | ≥ 3 | 6 | 17351.24 | 0.35 (0.12–0.63) | 1 | 35156.91 | 0.03 (0.00–0.09) | 12.16 (1.46–101.00) |
| BMI (kg/m^2^) | < 18.5 | 1 | 8354.52 | 0.12 (0.00–0.36) | 6 | 15193.74 | 0.39 (0.13–0.72) | 0.30 (0.04–2.52) |
|  | 18.5 to < 25 | 52 | 121332.20 | 0.43 (0.31–0.55) | 39 | 248408.96 | 0.16 (0.11–0.21) | 2.73 (1.80–4.13) |
|  | ≥ 25 | 11 | 56108.61 | 0.20 (0.09–0.32) | 14 | 108756.84 | 0.13 (0.06–0.20) | 1.52 (0.69–3.35) |
| Total cholesterol (mg/dL) | < 200 | 35 | 112227.51 | 0.31 (0.21–0.42) | 37 | 227176.13 | 0.16 (0.11–0.22) | 1.91 (1.21–3.04) |
|  | ≥ 200 | 29 | 73453.58 | 0.39 (0.26–0.54) | 22 | 144970.41 | 0.15 (0.09–0.22) | 2.60 (1.49–4.53) |
| Income | Low | 29 | 68648.82 | 0.42 (0.28–0.58) | 21 | 138391.96 | 0.15 (0.09–0.22) | 2.78 (1.59–4.88) |
|  | High | 35 | 117212.39 | 0.30 (0.20–0.40) | 38 | 234095.61 | 0.16 (0.11–0.22) | 1.84 (1.16–2.91) |

(E) Adjustment disorder

|  | | **Case cohort**  **(n = 29,172)** | | | **Reference cohort**  **(n = 58,344)** | | | **IRR**  **(95% CI)** |
| --- | --- | --- | --- | --- | --- | --- | --- | --- |
|  |  | **Cases** | **Person-years** | **IR per 1000 person-years (95% CI)** | **Cases** | **Person-years** | **IR per 1000 person-years (95% CI)** |  |
| Age (years) | < 60 | 549 | 138039.85 | 3.98 (3.65–4.31) | 604 | 277975.18 | 2.17 (2.00–2.35) | 1.83 (1.63–2.05) |
|  | ≥ 60 | 224 | 44618.00 | 5.02 (4.37–5.69) | 225 | 89961.66 | 2.50 (2.18–2.83) | 2.01 (1.67–2.42) |
| Sex | Male | 298 | 89648.81 | 3.32 (2.96–3.70) | 302 | 180555.55 | 1.67 (1.48–1.87) | 1.99 (1.69–2.33) |
|  | Female | 475 | 93009.04 | 5.11 (4.66–5.57) | 527 | 187381.30 | 2.81 (2.57–3.05) | 1.82 (1.60–2.06) |
| Sex & Age (years) | Male, < 60 | 215 | 70023.38 | 3.07 (2.67–3.48) | 232 | 141007.84 | 1.65 (1.44–1.86) | 1.87 (1.55–2.25) |
|  | Male, ≥ 60 | 83 | 19625.43 | 4.23 (3.36–5.15) | 70 | 39547.71 | 1.77 (1.37–2.20) | 2.39 (1.74–3.28) |
|  | Female, < 60 | 334 | 68016.47 | 4.91 (4.40–5.44) | 372 | 136967.34 | 2.72 (2.45–2.99) | 1.81 (1.56–2.10) |
|  | Female, ≥ 60 | 141 | 24992.57 | 5.64 (4.72–6.60) | 155 | 50413.96 | 3.07 (2.60–3.57) | 1.83 (1.46–2.31) |
| Smoking status | Yes | 101 | 35265.00 | 2.86 (2.33–3.43) | 116 | 71801.49 | 1.62 (1.32–1.92) | 1.77 (1.36–2.31) |
|  | No | 571 | 121119.52 | 4.71 (4.33–5.10) | 621 | 244441.89 | 2.54 (2.34–2.74) | 1.86 (1.66–2.08) |
|  | Ex-Smoking | 64 | 15293.41 | 4.18 (3.20–5.23) | 60 | 30832.22 | 1.95 (1.46–2.46) | 2.15 (1.51–3.06) |
| Frequency of alcohol consumption (per week) | 0 | 483 | 97253.56 | 4.97 (4.52–5.42) | 507 | 196220.96 | 2.58 (2.36–2.81) | 1.92 (1.70–2.18) |
|  | 1–2 | 213 | 64241.95 | 3.32 (2.88–3.77) | 249 | 129279.57 | 1.93 (1.69–2.17) | 1.72 (1.43–2.07) |
|  | ≥ 3 | 59 | 17135.57 | 3.44 (2.57–4.38) | 51 | 34161.46 | 1.49 (1.11–1.90) | 2.31 (1.59–3.35) |
| BMI (kg/m^2^) | < 18.5 | 34 | 8180.57 | 4.16 (2.81–5.62) | 30 | 15675.44 | 1.91 (1.28–2.62) | 2.17 (1.33–3.55) |
|  | 18.5 to < 25 | 517 | 119305.84 | 4.33 (3.96–4.71) | 582 | 244894.27 | 2.38 (2.18–2.57) | 1.82 (1.62–2.05) |
|  | ≥ 25 | 221 | 55107.72 | 4.01 (3.48–4.55) | 217 | 107223.10 | 2.02 (1.76–2.29) | 1.98 (1.64–2.39) |
| Total cholesterol (mg/dL) | < 200 | 454 | 110334.52 | 4.11 (3.74–4.50) | 492 | 224005.51 | 2.20 (2.00–2.39) | 1.87 (1.65–2.13) |
|  | ≥ 200 | 317 | 72162.80 | 4.39 (3.92–4.88) | 337 | 143489.44 | 2.35 (2.10–2.60) | 1.87 (1.60–2.18) |
| Income | Low | 258 | 67652.96 | 3.81 (3.36–4.29) | 281 | 137308.64 | 2.05 (1.81–2.29) | 1.86 (1.57–2.21) |
|  | High | 515 | 115004.89 | 4.48 (4.10–4.87) | 548 | 230628.20 | 2.38 (2.18–2.58) | 1.88 (1.67–2.13) |

(F) Organic mental disorders

|  | | **Case cohort**  **(n = 29,269)** | | | **Reference cohort**  **(n = 58,538)** | | | **IRR**  **(95% CI)** |
| --- | --- | --- | --- | --- | --- | --- | --- | --- |
|  |  | **Cases** | **Person-years** | **IR per 1000 person-years (95% CI)** | **Cases** | **Person-years** | **IR per 1000 person-years (95% CI)** |  |
| Age (years) | < 60 | 214 | 140175.31 | 1.53 (1.33–1.73) | 267 | 281005.55 | 0.95 (0.84–1.06) | 1.61 (1.34–1.92) |
|  | ≥ 60 | 524 | 44184.02 | 11.86 (10.86–12.88) | 771 | 89123.30 | 8.65 (8.05–9.27) | 1.37 (1.23–1.53) |
| Sex | Male | 277 | 90272.88 | 3.07 (2.71–3.43) | 341 | 181216.32 | 1.88 (1.68–2.09) | 1.63 (1.39–1.91) |
|  | Female | 461 | 94086.45 | 4.90 (4.45–5.36) | 697 | 188912.53 | 3.69 (3.42–3.96) | 1.33 (1.18–1.49) |
| Sex & Age (years) | Male, < 60 | 73 | 70898.04 | 1.03 (0.80–1.27) | 82 | 142154.86 | 0.58 (0.46–0.70) | 1.78 (1.30–2.45) |
|  | Male, ≥ 60 | 204 | 19374.84 | 10.53 (9.08–11.97) | 259 | 39061.46 | 6.63 (5.84–7.45) | 1.59 (1.32–1.91) |
|  | Female, < 60 | 141 | 69277.27 | 2.04 (1.70–2.38) | 185 | 138850.68 | 1.33 (1.15–1.53) | 1.53 (1.23–1.90) |
|  | Female, ≥ 60 | 320 | 24809.18 | 12.90 (11.49–14.35) | 512 | 50061.85 | 10.23 (9.35–11.13) | 1.26 (1.10–1.45) |
| Smoking status | Yes | 83 | 35598.50 | 2.33 (1.85–2.84) | 92 | 72361.62 | 1.27 (1.02–1.53) | 1.83 (1.36–2.47) |
|  | No | 568 | 122250.59 | 4.65 (4.27–5.03) | 823 | 246598.92 | 3.34 (3.11–3.57) | 1.39 (1.25–1.55) |
|  | Ex-Smoking | 55 | 15412.63 | 3.57 (2.66–4.54) | 79 | 30207.86 | 2.62 (2.05–3.21) | 1.36 (0.97–1.93) |
| Frequency of alcohol consumption (per week) | 0 | 524 | 98070.67 | 5.34 (4.89–5.80) | 784 | 198038.58 | 3.96 (3.69–4.24) | 1.35 (1.21–1.51) |
|  | 1–2 | 127 | 64996.78 | 1.95 (1.62–2.31) | 157 | 129147.49 | 1.22 (1.03–1.41) | 1.61 (1.27–2.03) |
|  | ≥ 3 | 71 | 17233.00 | 4.12 (3.19–5.11) | 75 | 34916.35 | 2.15 (1.69–2.63) | 1.92 (1.39–2.65) |
| BMI (kg/m^2^) | < 18.5 | 19 | 8315.05 | 2.29 (1.32–3.37) | 23 | 15304.35 | 1.50 (0.91–2.16) | 1.52 (0.83–2.79) |
|  | 18.5 to < 25 | 477 | 120408.67 | 3.96 (3.61–4.32) | 670 | 247785.53 | 2.70 (2.50–2.91) | 1.47 (1.30–1.65) |
|  | ≥ 25 | 242 | 55569.74 | 4.35 (3.82–4.91) | 345 | 106973.36 | 3.23 (2.89–3.57) | 1.35 (1.15–1.59) |
| Total cholesterol (mg/dL) | < 200 | 431 | 111359.24 | 3.87 (3.51–4.24) | 542 | 225750.98 | 2.40 (2.20–2.60) | 1.61 (1.42–1.83) |
|  | ≥ 200 | 305 | 72825.87 | 4.19 (3.72–4.67) | 493 | 143939.87 | 3.43 (3.13–3.73) | 1.22 (1.06–1.41) |
| Income | Low | 264 | 68132.18 | 3.87 (3.42–4.34) | 377 | 136835.35 | 2.76 (2.48–3.04) | 1.41 (1.20–1.65) |
|  | High | 474 | 116227.15 | 4.08 (3.72–4.45) | 661 | 233293.50 | 2.83 (2.62–3.05) | 1.44 (1.28–1.62) |

(G) Schizophrenia

|  | | **Case cohort**  **(n = 29,394)** | | | **Reference cohort**  **(n = 58,788)** | | | **IRR**  **(95% CI)** |
| --- | --- | --- | --- | --- | --- | --- | --- | --- |
|  |  | **Cases** | **Person-years** | **IR per 1000 person-years (95% CI)** | **Cases** | **Person-years** | **IR per 1000 person-years (95% CI)** |  |
| Age (years) | < 60 | 72 | 140239.72 | 0.51 (0.40–0.63) | 112 | 280994.90 | 0.40 (0.33–0.47) | 1.29 (0.96–1.73) |
|  | ≥ 60 | 82 | 45158.59 | 1.82 (1.44–2.21) | 125 | 90718.11 | 1.38 (1.15–1.62) | 1.32 (1.00–1.74) |
| Sex | Male | 74 | 90597.54 | 0.82 (0.64–1.00) | 111 | 181758.34 | 0.61 (0.50–0.73) | 1.34 (1.00–1.79) |
|  | Female | 80 | 94800.76 | 0.84 (0.66–1.03) | 126 | 189954.67 | 0.66 (0.55–0.78) | 1.27 (0.96–1.68) |
| Sex & Age (years) | Male, < 60 | 34 | 70852.96 | 0.48 (0.32–0.65) | 54 | 142088.32 | 0.38 (0.28–0.49) | 1.26 (0.82–1.94) |
|  | Male, ≥ 60 | 40 | 19744.59 | 2.03 (1.42–2.68) | 57 | 39670.03 | 1.44 (1.08–1.81) | 1.41 (0.94–2.11) |
|  | Female, < 60 | 38 | 69386.76 | 0.55 (0.37–0.74) | 58 | 138906.59 | 0.42 (0.32–0.53) | 1.31 (0.87–1.97) |
|  | Female, ≥ 60 | 42 | 25414.00 | 1.65 (1.18–2.16) | 68 | 51048.08 | 1.33 (1.02–1.67) | 1.24 (0.84–1.82) |
| Smoking status | Yes | 35 | 35625.86 | 0.98 (0.67–1.32) | 36 | 72254.92 | 0.50 (0.35–0.66) | 1.97 (1.24–3.14) |
|  | No | 97 | 123188.54 | 0.79 (0.63–0.95) | 173 | 247691.75 | 0.70 (0.60–0.80) | 1.13 (0.88–1.45) |
|  | Ex-Smoking | 7 | 15456.46 | 0.45 (0.13–0.84) | 18 | 31235.81 | 0.58 (0.32–0.86) | 0.79 (0.33–1.88) |
| Frequency of alcohol consumption (per week) | 0 | 98 | 98888.38 | 0.99 (0.80–1.19) | 173 | 199058.70 | 0.87 (0.74–1.00) | 1.14 (0.89–1.46) |
|  | 1–2 | 34 | 65102.76 | 0.52 (0.35–0.71) | 46 | 131119.86 | 0.35 (0.25–0.46) | 1.49 (0.96–2.32) |
|  | ≥ 3 | 12 | 17344.71 | 0.69 (0.35–1.10) | 13 | 33836.46 | 0.38 (0.18–0.62) | 1.80 (0.82–3.95) |
| BMI (kg/m^2^) | < 18.5 | 3 | 8355.11 | 0.36 (0.00–0.84) | 11 | 15337.16 | 0.72 (0.33–1.17) | 0.50 (0.14–1.79) |
|  | 18.5 to < 25 | 98 | 121125.70 | 0.81 (0.65–0.97) | 148 | 247821.62 | 0.60 (0.50–0.69) | 1.35 (1.05–1.75) |
|  | ≥ 25 | 53 | 55851.63 | 0.95 (0.70–1.22) | 78 | 108503.48 | 0.72 (0.56–0.88) | 1.32 (0.93–1.87) |
| Total cholesterol (mg/dL) | < 200 | 90 | 111960.77 | 0.80 (0.64–0.97) | 125 | 226297.02 | 0.55 (0.46–0.65) | 1.46 (1.11–1.91) |
|  | ≥ 200 | 64 | 73257.42 | 0.87 (0.67–1.09) | 112 | 145122.03 | 0.77 (0.63–0.92) | 1.13 (0.83–1.54) |
| Income | Low | 57 | 68520.34 | 0.83 (0.63–1.05) | 94 | 138346.29 | 0.68 (0.54–0.82) | 1.22 (0.88–1.70) |
|  | High | 97 | 116877.96 | 0.83 (0.67–1.00) | 143 | 233366.72 | 0.61 (0.51–0.72) | 1.35 (1.05–1.75) |

(H) Alcohol use disorder

|  | | **Case cohort**  **(n = 29,360)** | | | **Reference cohort**  **(n = 58,720)** | | | **IRR**  **(95% CI)** |
| --- | --- | --- | --- | --- | --- | --- | --- | --- |
|  |  | **Cases** | **Person-years** | **IR per 1000 person-years (95% CI)** | **Cases** | **Person-years** | **IR per 1000 person-years (95% CI)** |  |
| Age (years) | < 60 | 169 | 139810.84 | 1.21 (1.03–1.39) | 216 | 280402.61 | 0.77 (0.67–0.87) | 1.57 (1.28–1.92) |
|  | ≥ 60 | 49 | 45241.97 | 1.08 (0.80–1.39) | 106 | 90843.42 | 1.17 (0.95–1.40) | 0.93 (0.66–1.30) |
| Sex | Male | 165 | 90045.19 | 1.83 (1.55–2.12) | 253 | 181000.02 | 1.40 (1.23–1.57) | 1.31 (1.08–1.59) |
|  | Female | 53 | 95007.63 | 0.56 (0.41–0.72) | 69 | 190246.01 | 0.36 (0.28–0.45) | 1.54 (1.08–2.20) |
| Sex & Age (years) | Male, < 60 | 120 | 70384.04 | 1.70 (1.41–2.02) | 159 | 141390.45 | 1.12 (0.95–1.30) | 1.52 (1.20–1.92) |
|  | Male, ≥ 60 | 45 | 19661.15 | 2.29 (1.63–3.00) | 94 | 39609.56 | 2.37 (1.89–2.85) | 0.96 (0.68–1.38) |
|  | Female, < 60 | 49 | 69426.81 | 0.71 (0.52–0.91) | 57 | 139012.16 | 0.41 (0.31–0.52) | 1.72 (1.17–2.52) |
|  | Female, ≥ 60 | 4 | 25580.82 | 0.16 (0.04–0.31) | 12 | 51233.86 | 0.23 (0.12–0.37) | 0.67 (0.22–2.07) |
| Smoking status | Yes | 106 | 35201.75 | 3.01 (2.44–3.61) | 143 | 72912.43 | 1.96 (1.65–2.29) | 1.54 (1.19–1.97) |
|  | No | 69 | 123327.98 | 0.56 (0.43–0.70) | 120 | 246348.61 | 0.49 (0.40–0.58) | 1.15 (0.85–1.54) |
|  | Ex-Smoking | 32 | 15416.99 | 2.08 (1.36–2.85) | 43 | 31039.92 | 1.39 (1.00–1.80) | 1.50 (0.95–2.37) |
| Frequency of alcohol consumption (per week) | 0 | 46 | 99177.22 | 0.46 (0.33–0.60) | 94 | 197838.00 | 0.48 (0.38–0.57) | 0.98 (0.69–1.39) |
|  | 1–2 | 94 | 64799.23 | 1.45 (1.16–1.74) | 100 | 130997.23 | 0.76 (0.62–0.92) | 1.90 (1.43–2.52) |
|  | ≥ 3 | 75 | 16996.23 | 4.41 (3.47–5.41) | 123 | 34592.62 | 3.56 (2.95–4.19) | 1.24 (0.93–1.65) |
| BMI (kg/m^2^) | < 18.5 | 11 | 8316.47 | 1.32 (0.60–2.16) | 16 | 15782.43 | 1.01 (0.57–1.52) | 1.30 (0.61–2.81) |
|  | 18.5 to < 25 | 145 | 120818.44 | 1.20 (1.01–1.40) | 219 | 245390.54 | 0.89 (0.77–1.01) | 1.34 (1.09–1.66) |
|  | ≥ 25 | 62 | 55852.03 | 1.11 (0.84–1.40) | 87 | 109974.98 | 0.79 (0.63–0.96) | 1.40 (1.01–1.94) |
| Total cholesterol (mg/dL) | < 200 | 136 | 111697.01 | 1.22 (1.02–1.42) | 204 | 226232.18 | 0.90 (0.78–1.03) | 1.35 (1.09–1.68) |
|  | ≥ 200 | 81 | 73178.70 | 1.11 (0.87–1.35) | 118 | 144698.81 | 0.82 (0.67–0.97) | 1.36 (1.02–1.80) |
| Income | Low | 85 | 68370.42 | 1.24 (0.98–1.52) | 121 | 137783.05 | 0.88 (0.73–1.04) | 1.42 (1.07–1.87) |
|  | High | 133 | 116682.40 | 1.14 (0.95–1.34) | 201 | 233462.98 | 0.86 (0.75–0.98) | 1.32 (1.06–1.65) |

Abbreviations: IR = incidence rate; IRR = incidence rate ratio

**Figure S1.** Kaplan-Meier curve of psychiatric disease-free survival in patients with intestinal infectious disease (IID) and matched controls.


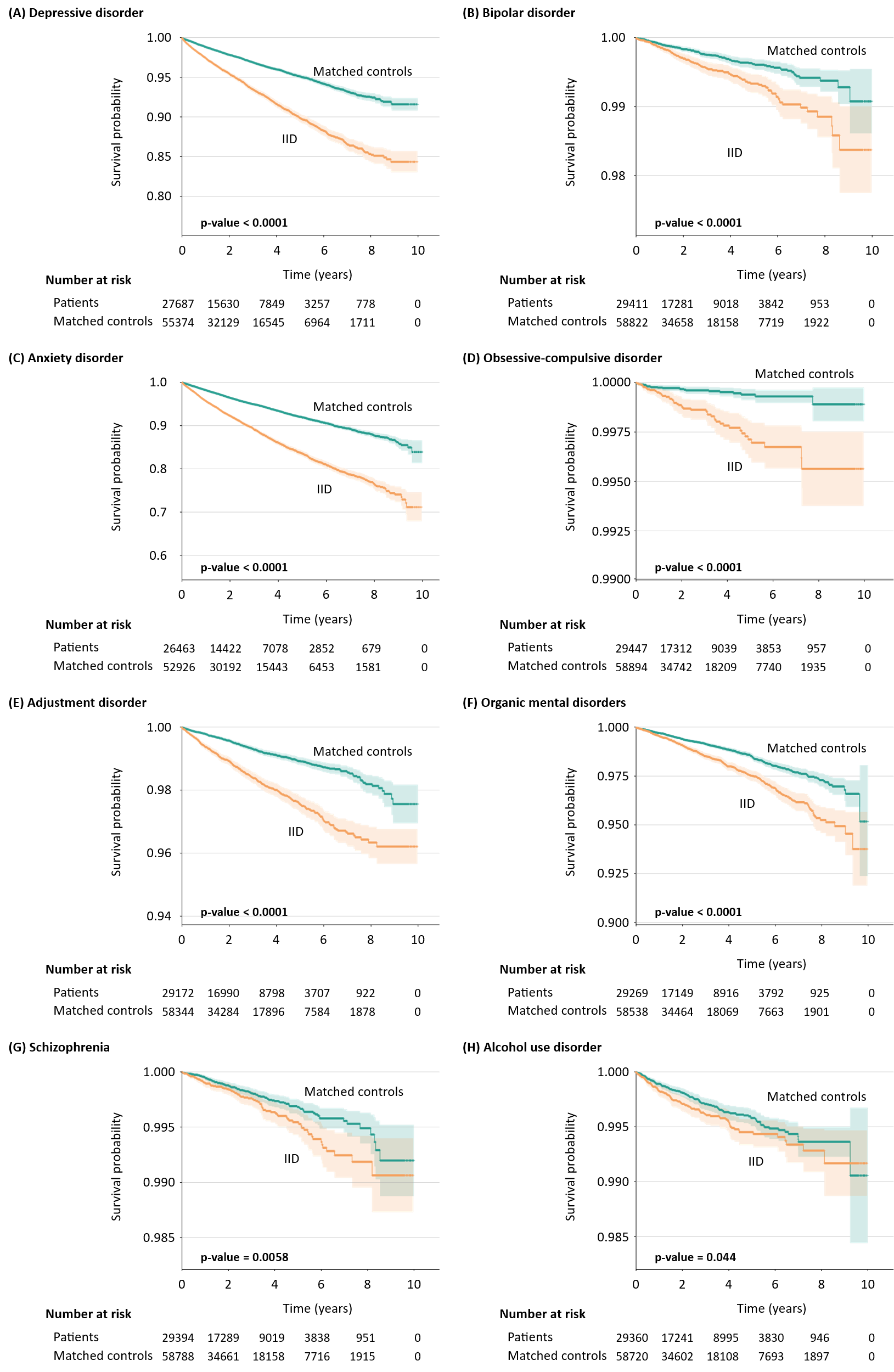

Supplement: Supplementary file 1 — Supporting Information S1 [file SMI-41-e70103-s001.docx]
